# Ligand Exchange and Binding at the Surface of PbS Quantum Dots Quantified Using Multimodal Magnetic Resonance

**DOI:** 10.1021/acsnano.5c03943

**Published:** 2025-07-22

**Authors:** Veera Venkata Shravan Uppala, Christian Y. Dones Lassalle, Jennica E. Kelm, Andrew M. Camp, Marc A. ter Horst, Alan R. Esker, Jillian L. Dempsey, Louis A. Madsen

**Affiliations:** † Department of Chemistry and Macromolecules Innovation Institute, 1757 Virginia Polytechnic Institute and State University, Blacksburg, Virginia 24061, United States; ‡ Department of Chemistry, 2331 University of North Carolina, Chapel Hill, North Carolina 27599-3290, United States

**Keywords:** semiconductor nanocrystals, ligand binding, populations, kinetics, diffusion

## Abstract

Semiconductor quantum dots (QDs) show promise for various applications, including biological imaging and photovoltaics. QDs are typically stabilized by surface-bound ligands, which exhibit a dynamic binding equilibrium. This study combines nuclear magnetic resonance (NMR) spectroscopy and diffusometry to quantify the populations and kinetics of oleic acid (OAH) ligand binding to PbS QD surfaces. In addition to quantifying ligand population fractions in bound and free states, our analysis reveals the existence of a third ligand state, which we hypothesize to be the weak coordination of the OAH at (100) sites through the acidic headgroup (−COOH). Thus, bound ligands exist in two subpopulations: weakly bound OAH on (100) facets and strongly bound oleate (OA) on (111) facets. Through variations in temperature and concentration, we assess the changes in ligand populations in different states and determine the energetics of their exchange equilibria. Additionally, using dynamic NMR spectroscopy, we quantify rapid exchange rates (0.09–2 ms) between weakly bound and free OAH ligands as a function of OAH titration concentration and temperature. These findings highlight the complexity of ligand binding mechanisms and enable strategies for precisely tuning QD surface properties, with significant implications for the innovation of next-generation optoelectronic materials.

## Graphical abstract

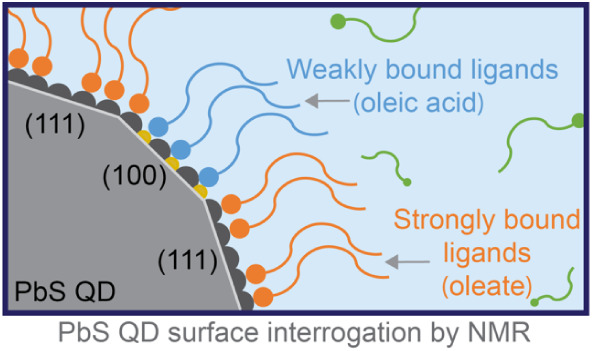

Semiconductor quantum dots (QDs) have garnered significant attention due to their remarkable size-tunable optoelectronic properties. These nanocrystals have been extensively investigated for their potential in diverse applications, including photovoltaic cells, light-emitting diodes (LEDs), and biological cell imaging.^1,2^ The unique optical and electronic properties of QDs stem from quantum confinement effects and can be precisely tailored by adjusting the size of the nanocrystal. However, the integration of QDs into optoelectronic devices heavily depends on the nature of their surface, a consequence of their high surface-to-volume ratio. The surface composition significantly influences optical and electronic properties of QDs, driving extensive research into QD surface chemistry.^3–8^ Therefore, understanding surface passivation and ligand interactions is crucial to optimize QD properties for targeted applications.

Tuning the QD surface for desired applications requires knowledge of the inorganic crystal lattice, the organic ligand shell, and the interface between these two components. The inorganic component of the QD can vary in crystal structure, faceting, and morphology.^3,6,9–11^ Additionally, the surface ions may vary in the oxidation state and coordination number. The organic ligands that interface with surface ions vary in binding motif, as defined by Green’s covalent bond classification and coordination geometry, which influences the binding energy between the ligand and the surface. Green’s covalent bond classification categorizes ligands as X-type, L-type, or Z-type.^12,13^ X-type ligands are anionic and compensate for excess cationic charge by donating one electron to the surface metal cation. X-type ligands include carboxylates, such as oleate (OA), and thiolates. L-type ligands are neutral two-electron donors and generally do not impact the QD charge. Amines and phosphines are examples of L-type ligands, although carboxylic acids (e.g., oleic acid) and thiols can also bind to the QD surface as L-type ligands.^3^ Z-type ligands are neutral two-electron acceptors and coordinate to surface chalcogen anions. Z-type ligands coordinated to the QD surface are typically classified as a metal with two anionic X-type ligands attached to it, such as Pb­(OA)_2_ and Cd­(OA)_2_.

Ligand exchange reactions, commonly employed to alter either QD solubility or the functionality of the QD surface, can vary depending on the surface anchoring group and chain length. The simplest example of ligand exchange is that between ligands with the same binding group. For instance, carboxylic acids can exchange with native X-type carboxylate ligands through an acid–base mechanism. This exchange reactivity results in the liberation of natively bound OA in the form of oleic acid (OAH) and the binding of the non-native carboxylate to the surface as an X-type carboxylate ligand.^14,15^ A similar mechanism can occur through a carboxylate salt, leading to liberation of X-type OA with the corresponding counterion and binding of the exchanging X-type carboxylate to the surface.^8,16^ However, when the exchange ligand and the native ligand differ in binding group, complex and potentially multimechanism exchange reactions can result, as evidenced by studies of L-type amines and thiols reacted with OA-capped PbS QDs.^3,17^

Controlled tunability of the QD surface requires both a mechanistic understanding of ligand exchange and a quantitative technique to monitor the process. Several studies have utilized techniques like photoluminescence spectroscopy, 1D ^1^H NMR spectroscopy, and nuclear Overhauser effect spectroscopy (NOESY)^18–21^ to monitor reactivity and decipher reaction mechanisms. Among these, 1D ^1^H NMR spectroscopy is arguably the most popular technique to quantify surface reactivity due to the unique line shape of the surface-bound ligand signal.^13–15,22–24^

In most ligand exchange mechanisms, the exchange between the native and the non-native ligands is assumed to rely on a two-state system where ligands are exclusively bound to the surface or freely diffusing in solution.^14,15,25,26^ In this scenario, the exchange proceeds through a one-step process of non-native ligand binding coupled with native ligand dissociation. However, several studies have found evidence for a third state beyond the classically defined bound and free states.^6,27–32^ In 2010, work by Fritzinger et al. proposed a two-step exchange mechanism where free/exchanging ligands are “physisorbed” to the QD surface before “chemisorption” to replace the native ligand.^29^ In 2014, Valdez et al. leveraged diffusion-ordered spectroscopy (DOSY) and 1D ^1^H NMR spectroscopy to identify and quantify three dodecylamine (DDA) ligand populations in ZnO nanocrystals: strongly bound/weakly associated ligands with diffusion coefficients of 1 × 10^–10^ m^2^·s^–1^ and free ligands with diffusion coefficient of 1.45 × 10^–9^ m^2^·s^–1^.^6^ By titrating excess DDA and monitoring diffusion changes, they revealed a dynamic equilibrium between weakly associated and free ligands and attributed a low density of strongly bound ligands to native surface hydroxides. In 2019, Weir and co-workers found greater-than-monolayer coverage of oleic acid (OAH) on PbS QDs with small-angle X-ray and neutron scattering, suggesting the presence of physisorbed ligands.^28^ The following year, Liu et al. utilized a suite of spectroscopic techniques to propose a “two-step ligand exchange” and related the physisorption of ligands to van der Waals interactions.^30^ While a third state has been identified in several reports, a full understanding of the identity of the three states, their interplay, and their complete quantification has remained elusive.

Clearly, ligand exchange reactions at the QD surface can be more complex than a two-state model, and three-state systems require further investigation. PbS QDs serve as an excellent model system to study surface reactivity due to their air stability, low preparation cost, and widely tunable bandgap in the near-infrared region, making them promising for photovoltaic and photodetector applications.^33,34^ Moreover, the X-type ligand exchange of OA-capped PbS QDs with carboxylic acids has been well-studied and is reported to occur through an acid–base mechanism.^8,14,25^ This results in a system with simplified reactivity, thus avoiding the complexities of the multimechanistic reactivity previously reported for other exchange ligands.

The limited understanding of ligand binding and exchange mechanisms requires continued development of simpler and more robust characterization methods. To address this challenge, we investigated the ligand dynamics of OA-capped PbS QDs using multimodal NMR techniques, including diffusometry and 1D ^1^H spectroscopy, to quantify the populations of free and surface-bound ligands (Figure 1). This quantification approach was previously applied by our group to block copolymer micelle systems.^35–37^ Our quantitative analysis reveals the existence of a third ligand state, in addition to bound and free ligands, upon the titration of excess OAH into a solution of OA-capped PbS QDs. This analysis further demonstrates a ligand binding mechanism that is more complex than the previously accepted two-state model.

**Figure 1:**
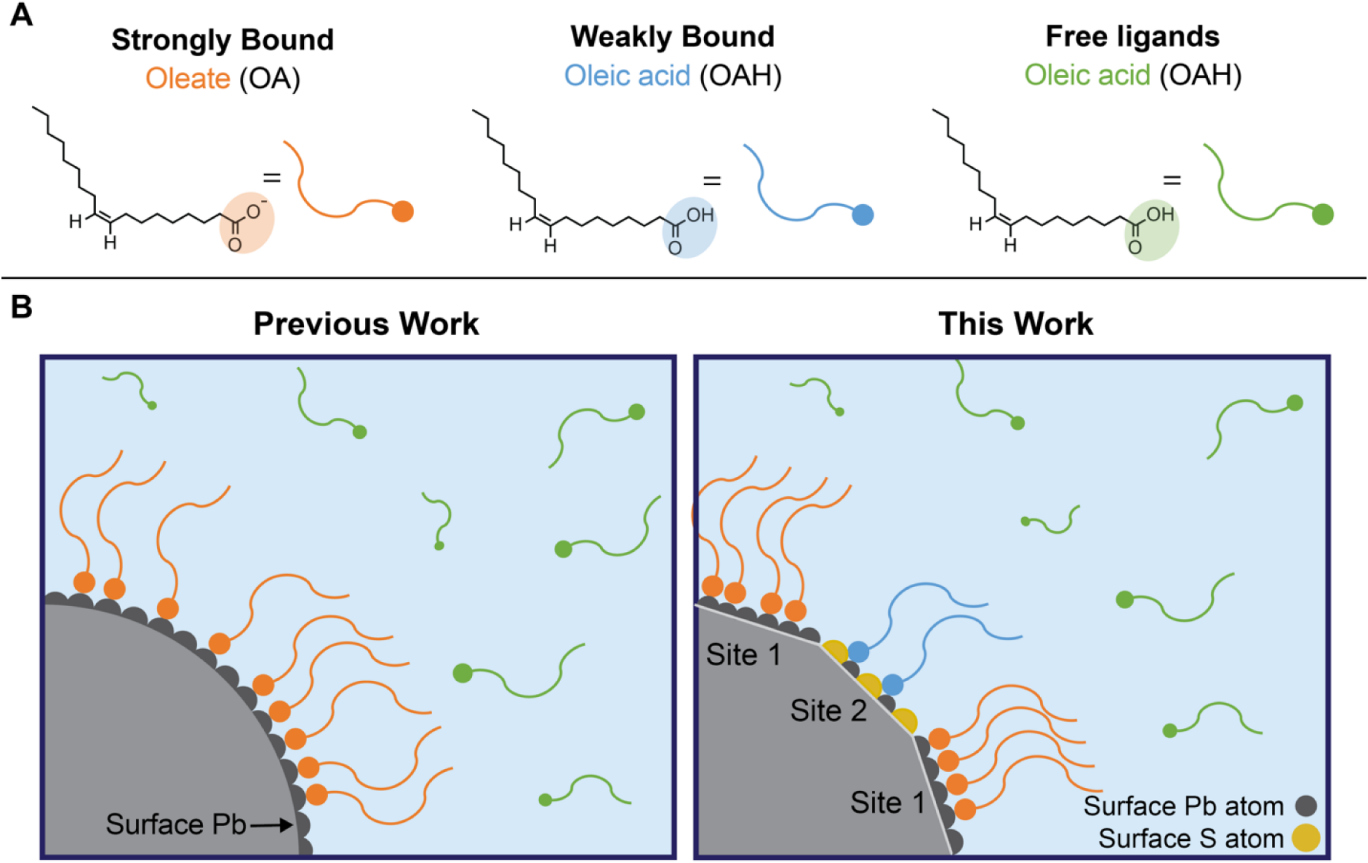
(A) Chemical structures of the OAH and OA ligands employed and quantified in this work. (B) Summary of ligand-based reactions that assume only bound and free ligands (left) compared to our facet-specific model with *S_bound*, *W_bound*, and free ligands (right).

In addition to the free state of OAH and the OA strongly bound as an X-type ligand to Pb-rich (111) facets, we propose that this third ligand state corresponds to weak OAH coordination to Pb and S atoms on the (100) facets of the PbS QD surface. Importantly, OA ligands may also be coordinated to core Pb­(II) ions or to Pb­(II) ions that are part of Z-type ligands, further diversifying the binding environments present on the QD surface.^9^ These observations contrast with previous work summarized above, which proposes the third ligand state as an associated ligand through weak intermolecular interactions. As such, the classically defined “bound ligands” should be subcategorized into weakly bound (which we call *W_bound*) ligands and strongly bound (which we call *S_bound*) ligands, which differ in binding motifs and facet coordination.

Using NMR diffusometry and spectroscopy, we quantify the population fractions of *S_bound*, *W_bound*, and free ligands as a function of excess titrated OAH concentration and temperature. By associating these ligand populations with their respective facets, we provide a new understanding of the binding environments. Additionally, dynamic NMR spectroscopy (line shape analysis of 1D NMR as a function of temperature) provides quantification and insights into the exchange kinetics of ligands between states, broadening our understanding of the ligand exchange mechanism. These results enhance our understanding of ligand binding and exchange processes at QD surfaces, facilitating more informed strategies for surface modification to achieve desired properties. The NMR techniques employed in this study are compatible with most standard spectrometers and adaptable to various chemical systems, enabling widespread application to study diverse QD-ligand interactions.

## Results and Discussion

### PbS QD Characterization

OA-capped PbS QDs were synthesized following a modified procedure from Hines and Scholes^3,17,38^ and were carefully purified through precipitation–centrifugation steps to remove the unbound, weakly bound, and unreacted oleate (OA) species, as judged by the ^1^H NMR spectrum. In other words, we detect bound OA at 24 mM with a signal-to-noise ratio SNR ≈ 700 and full width half maximum (fwhm) = 60 Hz, with the number of scans used being 8. Thus, the limit of detection (SNR = 2) for free OA (fwhm ∼1 Hz) in the NMR spectrum is ∼1 μM, meaning that any remaining OA (or other small molecule) species after purification, which are not visible in the NMR spectrum, must be present at less than that concentration. ^1^H NMR spectroscopy was used to determine the QD-bound ligand density by integrating the alkene resonances of bound OA and comparing them to a known concentration of ferrocene standard internal to the sample solution. The total ligand coverage is 158 OA per QD, corresponding to a packing density of 3.9 ligands/nm^2^ using a quasi-spherical approximation. These ligands are traditionally considered strongly bound, chemisorbed species, as discussed below.^39^ UV–vis absorbance spectroscopy studies revealed a maximum absorption at 913 nm (Figure S2), corresponding to a diameter of 3.0 ± 0.2 nm, in agreement with that determined by TEM (Figure S3). This particle diameter was further validated by NMR diffusometry, which yielded a diameter of 3.6 ± 0.4 nm. The particle size determined through diffusometry (a hydrodynamic diameter) is different from the ones determined through UV–vis and TEM but is within error (see discussion of particle size discrepancy in Sections S1 and S5). A QD size of 3.6 nm was used for all our quantitative analyses since it was determined through NMR diffusometry, the main technique used to probe ligand interactions in this work.

3.6 nm PbS QDs contain (100) and (111) facets, with their relative areas dependent on particle size, as theoretically predicted from the Wulff construction model and observed experimentally through the displacement of metal carboxylate complexes in differently sized QDs.^40–42^ Using a structural model of 3.6 nm PbS QDs generated in VESTA, and based on expected faceting for QDs of this size, we estimate a total of 306 Pb ions on the QD surface (see Section S6 for additional information on this estimation). This number includes Pb ions on the (111) and (100) facets as well as on edge sites.

### Evidence for Weakly Bound Ligands by 1D ^1^H NMR and NMR Diffusometry Techniques

To understand the dynamic behavior of the oleate (R-COO^–^, OA) ligand and its role in binding to PbS QDs, we first examine the ^1^H NMR spectrum of a control sample containing free oleic acid (R-COOH, OAH) ligand in toluene-d_8_ (Figure S1). OAH exhibits a distinct alkenyl ^1^H NMR peak, which we term *ν*_free_ (chemical shift of OAH), near 5.6 ppm that is well-resolved from the toluene-d_8_ solvent peak at 7.2 ppm, from the internal standard ferrocene at 4.1 ppm, and from the other alkyl proton signals of OAH below 2.4 ppm. The alkenyl ^1^H signal from this free OAH has fwhm = 7 Hz (Figure 2, black trace), which is characteristic of a small molecule undergoing rapid tumbling in solution. This well-resolved *ν*_free_ signal provides a clear “handle” for studying ligand dynamics.

**Figure 2:**
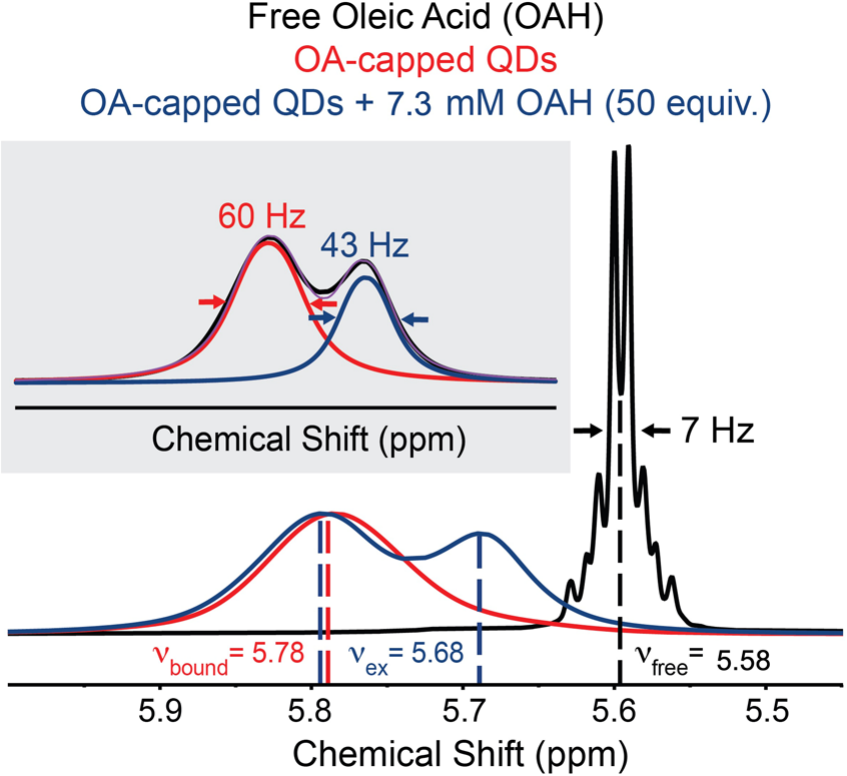
^1^H NMR spectra of free OAH (black), 150 μM 3.6 nm OA-capped PbS QDs (red), and 150 μM 3.6 nm OA-capped PbS QDs titrated with 50 equiv of OAH per QD (7.3 mM) in toluene-d_8_ (blue). These spectra highlight the alkenyl protons of the ligand. The gray inset shows the deconvoluted spectrum for 3.6 nm OA-capped PbS QDs titrated with 50 equiv of OAH per QD, giving us the fwhm for the bound and exchanging ligand signals. The spectra are scaled relative to the signal intensity of the *ν_S___bound_* peak (red).

We also examined a control sample containing 150 μM of 3.6 nm PbS QDs capped with OA ligands in toluene-d_8_. These OA ligands are known to be strongly bound to the QD (*S_bound*), and their alkenyl proton (*ν_S_bound_*) shifts downfield to 5.8 ppm due to an aromatic solvent-induced shift (ASIS),^43^ as shown by the red trace in Figure 2. This shift occurs because the aromatic solvent is excluded from the ligand shell, so these alkenyl protons do not experience the same aromatic environment as those of fully solvated free OAH ligands. Additionally, the *ν_S_bound_* signal broadened to fwhm = 60 Hz. We attribute this broadening to homogeneous line broadening in the NMR spectrum resulting from the restricted tumbling motion of the *S_bound* OA ligands relative to free ligands in solution.

As 7.3 mM of excess OAH (50 equiv per QD) is titrated into the OA-capped 3.6 nm PbS QD solution, we observe two broad alkenyl proton signals overlapping in the range of 5.5–6 ppm (Figure 2, blue trace). The first broad signal at 5.78 ppm has fwhm = 61 Hz (inset figure in Figure 2), which we assign as the *S_bound* OA ligand because its chemical shift and fwhm match the *S_bound* signal from the control sample (Figure 2, red trace). The second broad signal appears at 5.68 ppm, positioned between *ν_S_bound_* (5.78 ppm) and *ν*_free_ (5.58 ppm). We refer to this as an “intermediate” signal for now. Further deconvolution of the overlapping signal (inset in Figure 2) shows that this intermediate signal has fwhm = 43 Hz, which is broader than the free OAH signal (7 Hz) but narrower than the *S_bound* OA ligand signal (60 Hz).

As we increase the titrated concentration of excess (initially free) OAH (7.3 mM, 14.3 mM, and 27.2 mM), the intermediate signal shifts upfield from 5.68 ppm to 5.62 ppm, approaching the chemical shift of free OAH (*ν*_free_ = 5.58 ppm). Additionally, the intermediate signal’s fwhm narrows from 43 Hz to 33 Hz (see Figure S6 in Section S4). However, the chemical shift and width of the *S_bound* OA peak at 5.78 ppm does not change as OAH is titrated, signifying that the intermediate peak is not related to the QD-bound ligands that are on the surface at the start of the titration. This concentration-dependent shift indicates that the intermediate signal is instead influenced by the amount of free OAH ligands in solution.^40^

The trend in the chemical shift and width of the intermediate signal, lying between those of *S_bound* OA and free OAH ligands, indicates that it arises due to a fast exchange between OAH in two states. If the exchange between *S_bound* and free ligands is rapid compared to the relevant NMR time scale (3.9 ms, see concepts of NMR time scales detailed in Section S2), the two signals would coalesce into one peak (Figure S13). Instead, we observe a gradual upfield shift of the intermediate signal as more OAH is titrated, while the chemical shift and width of the *S_bound* OA signal remain unchanged. This also indicates that the exchange between *S_bound* OA and free OAH is much slower than the NMR time scale of measurement (τ_ex,bind_ ≫ 3.9 ms) and that the intermediate signal does not arise from exchange between *S_bound* OA and free OAH. Based on these data, we attribute the intermediate signal to the rapid exchange between free OAH and OAH that is weakly bound (*W_bound*) to the QD surface. The chemical shift of this intermediate signal, which we now term the exchanging signal (*ν*_ex_), therefore depends on the titration concentration of free OAH.

In Section S10, we explain in more detail how spectral changes depend on exchange rates and the time scale of NMR measurements. While simple 1D ^1^H NMR spectroscopy qualitatively estimates exchange rates, we describe quantitative methods in the “Kinetics” section below to determine these exchange rates.

To further confirm the fast exchange between free and *W_bound* OAH ligands, we employ NMR diffusometry with the PGSTE pulse sequence to probe diffusion rates of the ligands in different states. Figure 3 shows plots of the NMR signal intensity as a function of experimental parameters, which yield measured *D* values when fitted with eq 18 in the **Methods** section below. As expected, free OAH at 5.6 ppm (black trace) exhibits a faster diffusion coefficient, *D*_free_ = 7.1 ± 0.2 × 10^–10^ m^2^ ·s^–1^, compared to *S_bound* OA at 5.8 ppm, which has a much slower diffusion coefficient, *D_s_bound_* = 1.3 ± 0.1 × 10^–10^ m^2^·s^–1^, at 23 °C. The slower diffusion of the bound OA results from the larger hydrodynamic diameter of the entire colloidal particle. Since the species being probed (free OAH and *S_bound* OA) are each in a single diffusing environment, each set of experimental data fits well with a single diffusion component, as shown in Figure 3.

**Figure 3:**
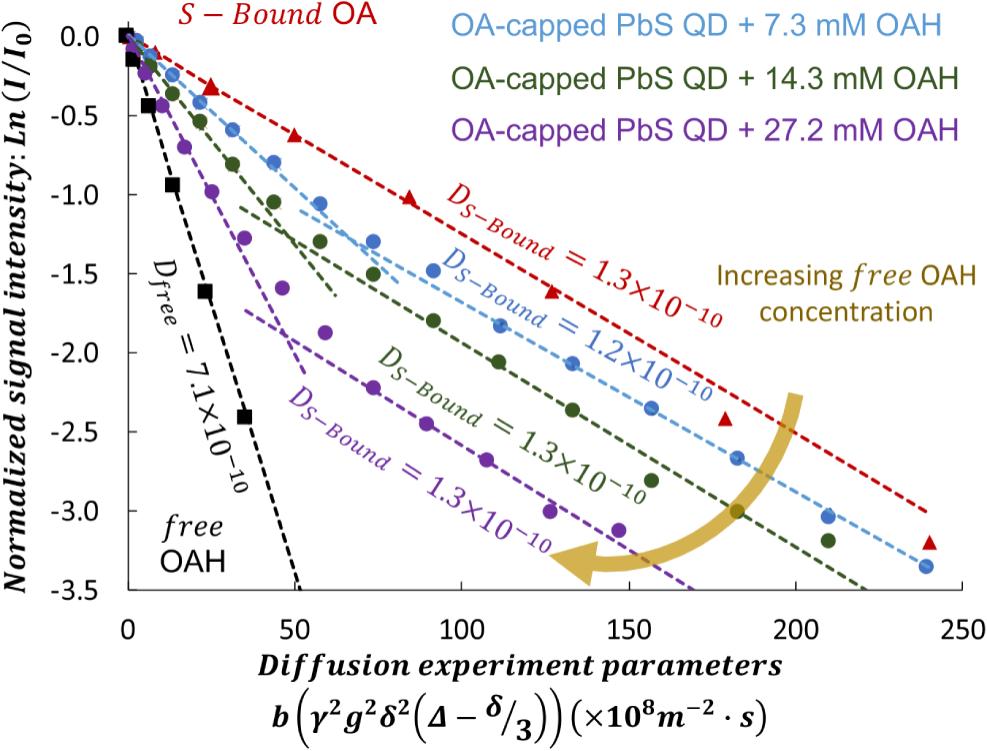
Variation in the attenuation of NMR signal intensity of the alkenyl proton signal of the OAH (integrated from 5.5 to 6 ppm) as a function of excess OAH concentration in a solution containing 150 μM, 3.6 nm OA-capped PbS QDs measured at 23 °C. *b* is the diffusometry parameter that contains all known NMR experimental parameters. The dashed lines provide guides to the eye and represent single- or two-component diffusion coefficients (lines are not actual fits to the experimental data to make for an easy slope comparison). Increasing the OAH concentration increases the *D* of the faster diffusing species, while the *D* for the slower diffusing species remains unchanged, highlighting that the slow-diffusing species are *S_bound* OA ligands in very slow exchange with free OAH (≫200 ms).

When we titrate an excess OAH (7.3 mM to 27.2 mM) into a solution containing OA-capped PbS QDs, we observe two diffusing species with unique diffusion rates, as evidenced by the two dashed lines with different slopes (Figure 3). For each of these two-component NMR signal intensity attenuation (Stejskal–Tanner) plots, the slower diffusing species has a diffusion coefficient equal to *D_S_bound_*. Since the dashed lines remain parallel to the OA-capped QD sample’s line, these species correspond to *S_bound* OA ligands, and they do not participate in observable exchange with the free ligands on the time scale of the NMR diffusometry measurement (see Section S2), which is equal to the diffusion time Δ = 25 ms. Table 1 lists *D* values for *S_bound* ligands for all samples, which are within errors. Additionally, increasing Δ to 200 ms causes no change to the OA alkenyl proton signal profile (fwhm and chemical shift), demonstrating that the exchange time τ_ex,*S_*b*ound*_ of the *S_bound* OA with free OAH is substantially longer than 200 ms.

**Table 1:** Population Fractions and Diffusion Coefficients of Ligands in All Three Distinct States^a^

Temperature (23 ̊C)	Population Fractions of Ligands				Ligand Diffusion Coefficients (/10^–10^m^2^·s^–1^)		
Sample	*f_S_bound_*	*f*_ex_	*f*_free_	*f_W_bound_*	*D_S_bound_*	*D*_ex_	*D*_free_
QD Only	1.00	0.0	0.0	0.0	1.3 ± 0.1	0.0	0.0
QD + 7.3 mM OAH	0.64	0.36	0.10	0.26	1.2 ± 0.1	2.9 ± 0.1	7.1 ± 0.2
QD + 14.3 mM OAH	0.46	0.54	0.32	0.22	1.3 ± 0.1	4.7 ± 0.2	7.1 ± 0.2
QD + 27.2 mM OAH	0.31	0.69	0.51	0.18	1.3 ± 0.1	5.4 ± 0.3	7.1 ± 0.2

a *f_S_bound_* and *f*_ex_ are from the *S_bound* and exchanging signals, and the fractions are derived by deconvoluting the ^1^H NMR spectrum and assuming *f_S_bound_* + *f*_ex_ = 1. *f*_free_ and *f_W_bound_* are determined from eq 2, and the constraint *f*_free_ + *f_W_bound_* = *f*_ex_. *D*_free_ is the diffusion coefficient of free ligands in solution and is the same in all samples because the concentrations in all samples are too low to change the viscosity of the solution. *D_S_bound_* is the diffusion coefficient of bound ligands determined by probing the bound signal *ν_S_bound_* at 5.78 ppm. We assume that for *W_bound* ligands that are instantaneously bound to the QD, *D_W_bound_* = *D_S_bound_*.

When the concentration of excess OAH titrated increases from 7.3 mM to 27.2 mM, the *D* of the faster diffusing species increases but remains slower than *D*_free_ (Figure 3 and Table 1). Since this diffusion coefficient (*D*_ex_) is faster than bound OA but slower than free OAH, we surmise that these intermediate diffusion coefficient values arise from the rapid averaging of the exchanging signals (τ_ex_ ≪ Δ) of the *W_bound* and free OAH ligands. With this direct evidence of three ligand states—*S_bound*, *W_bound*, and free—we quantify the fraction of ligands in each state.

### Quantifying Population Fractions of Ligands in Different States Using NMR Spectroscopy and Diffusometry

^1^H NMR spectroscopy enables quantification of the population fractions of *S_bound* ligands (*f_S_bound_*) and the fast-exchanging ligands (*f*_ex_) by deconvoluting the overlapping peaks that contribute to the total ligand content in the solution (*f_S_bound_* + *f*_ex_ = 1). To further quantify the population fractions of free and *W_bound* ligands that contribute to the exchanging signal, we use the diffusion coefficients and signal intensity fractions measured for the different ligand species (Table 1).

Given that the exchange between free and *W_bound* ligands is fast relative to the diffusion time (Δ), the measured *D*_ex_ is the weighted average of the diffusion coefficients for free (*D*_free_) and *W_bound* ligands (*D_W_bound_*). Using this relationship, we determined the population fractions of the free ligand (*f*_free_) and *W_bound* (*f_W_bound_*) ligand using eq 1:

1 $$D_{ex}=\frac{f_{free}}{f_{ex}}\times D_{free}+\frac{f_{\mathrm{W\_bound}}}{f_{ex}}\times D_{\mathrm{W\_bound}}$$

where the total population fraction of free and *W_bound* ligands is equal to the exchange signal’s population fraction (*f*_free_ + *f_W_bound_* = *f*_ex_). Although *W_bound* ligands have a different chemical binding motif with the QD surface compared to *S_bound* ligands (evident from the exchange rates with *free* ligands), it is reasonable to assume that the unknown *D_W_bound_* is equal to the measured *D_S_bound_* (Table 1) as they both represent the Brownian motion of the QD particle and thus exhibit similar average spin dynamics.

Since *ν*_ex_ depends on the population fraction and chemical shifts of *W_bound* and free OAH ligands in the solution, we can use eq 2 to determine the chemical shift of the *W_bound* ligand (*ν_W_bound_*) when the ligand instantaneously resides on the QD:

$$\nu_{ex}=\frac{f_{free}}{f_{ex}}\times \nu_{free}+\frac{f_{\mathrm{W\_bound}}}{f_{ex}}\times \nu_{\mathrm{W\_bound}}$$ 2

This determined *ν_W_bound_* is the same as *ν_S_bound_* for 7.3 mM, 14.3 mM, and 27.2 mM OAH concentrations (Table S6). This equivalence of the chemical shift for *W_bound* and *S_bound* (*ν*_S_bound_ ≈ *ν_W_bound_*) ligands suggests that the alkenyl signal magnetic environment is the same for both ligand environments, even though the ligand interactions with the QD surface differ. This chemical environment similarity further supports the assumption that the diffusion coefficient of an instantaneously *W_bound* ligand on the QD is equal to that of an instantaneously *S_bound* ligand, i.e., *D_W_bound_* = *D_S_bound_*. Table 1 lists the population fractions and diffusion coefficients of ligands in the three distinct states as a function of the titration of the OAH into OA-capped PbS QD solutions. These data show that as the OAH titration concentration increases, the fraction of free OAH increases, and thus, the chemical shift of the exchanging signal moves upfield toward free OAH.

Figure 4 conceptually summarizes our experimental observations, depicting ligands in three distinct states: *S_bound*, *W_bound*, and free. A fast exchange occurs between free and *W_bound* OAH ligands (relative to the 3.9 ms NMR peak exchange time scale), while the exchange between free OAH and *S_bound* OA ligands is far slower (much slower than Δ = 200 ms). We discuss and quantify ligand exchange rates further in the “Kinetics” section below. However, the population fractions alone do not provide a complete understanding of the occupancy of the ligands on the QD surface. Further quantification of ligand populations per QD enables deeper insights into the trends in ligand dynamics, which we discuss in the next section.

**Figure 4:**
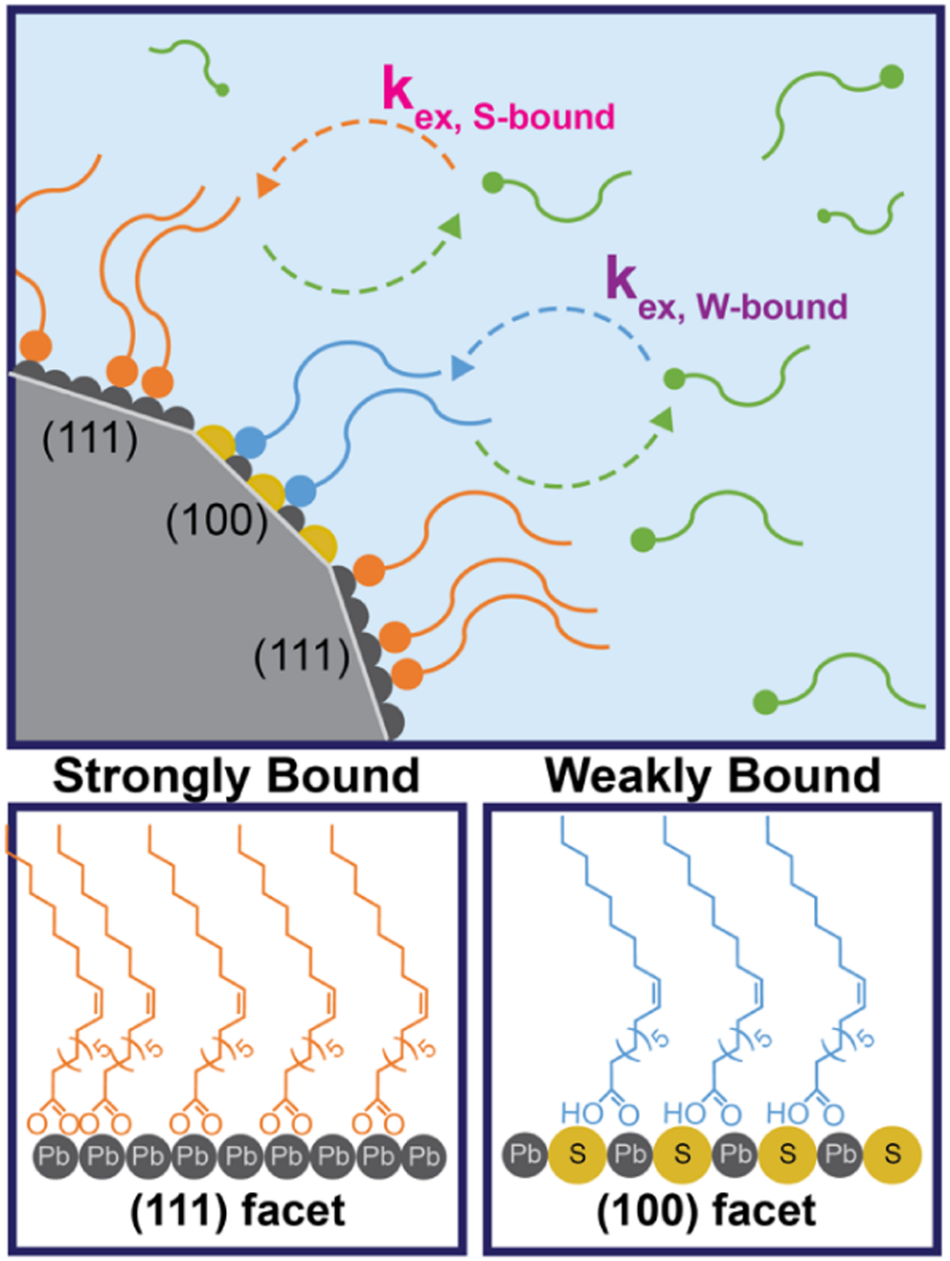
Illustration of facet-specific ligand interactions at the surface of the 3.6 nm PbS QDs. *S_bound* OA and *W_bound* OAH ligands that are instantaneously bound to the QD are assumed to have the same diffusion coefficients and chemical shifts (within errors), as supported by eqs 2 and 3.

### Equilibrium Constant Quantification Based on Proposed *W_Bound* Ligand Identity

As OA-capped PbS QDs are titrated with excess OAH, we propose that two processes occur simultaneously. First, free OAH ligands can form a strong covalent bond with surface Pb ions, resulting in *S_bound* OA ligands. Ab initio total energy calculations, reported by Wang et al.,^39^ suggest that these bound ligands coordinate to Pb ions at the (111) facet to neutralize their cationic charge.^34,44^ We find that the exchange time between the *S_bound* and free ligands is much longer than the 1D NMR measurement time scale in the spectrum, given by the inverse of the peak frequency difference (τ ∝ 1/δ*ν*, see Section S2). As a result, the bound signal chemical shift and fwhm remain unchanged in the NMR spectrum, even when excess OAH is titrated into the OA-capped PbS QD solution. The small change in the *S_bound* signal intensity (at 5.8 ppm) reflects the change in *S_bound* ligand population rather than a change in the exchange rate.

In a second process, we propose that *W_bound* ligands bind to chemically distinct sites on the QD surface. We envision three possible scenarios for the nature of the weak interaction of *W_bound* ligands with the QD surface: (1) van der Waals interactions between aliphatic ligand chains could trap them in a ligand shell, (2) hydrogen bonding could occur between ligands and surface hydroxides,^6^ or (3) OAH ligands can bind in a bridging-bidentate fashion to Pb and S ions on the (100) facet (Figure 4).^39^ While we cannot experimentally confirm the role surface hydroxides play in *W_bound* OAH binding, ab initio calculations by Wang et al. suggest that surface hydroxides form only at (111) facets.^39^ Furthermore, penetration of the OAH into the densely packed OA ligand shell in the (111) facet is sterically hindered, and thus, we rule out scenario (2).

To investigate the role of van der Waals interactions described in scenario (1), we conducted NMR diffusometry experiments using OA-capped QDs titrated with an aliphatic molecule incapable of chemical coordination to the QD surface. We selected 1-octadecene (ODE), which has a long-chain alkene structurally similar to OAH but lacks the carboxylic acid headgroup necessary for coordination to the QD surface. If *W_bound* ligands participate in van der Waals interactions alone, we would observe a measurable effect on ODE’s diffusion coefficient when titrated into a QD solution. However, upon titrating 100 equiv of ODE per QD (14.3 mM) into a solution of OA-capped PbS QDs, we observe no change in the diffusion coefficient of ODE compared to free ODE in the absence of QDs (see Section S3 for more details). The observed diffusion coefficient was identical when probed using the NMR signal from different protons of the ODE molecule. Additionally, we analyzed the acidic proton (−COOH) using 1D ^1^H NMR spectra at varying titration concentrations of excess OAH and temperature to better understand its dynamic behavior (see Section S14 for more details). Both findings strongly suggest that *W_bound* ligands coordinate with the QD surface through the −COOH headgroup rather than incorporation via van der Waals interactions. Future work will further test this hypothesis by quantifying variations in surface-bound ligand populations as a function of QD particle size, as the facet ratios in PbS QDs are known to be size-dependent.

Thus, we propose scenario (3) that *W_bound* ligands bind to the (100) facet. The dynamic equilibrium observed in the 1D ^1^H NMR spectra between *W_bound* and free OAH indicates that these ligands bind more weakly than *S_bound* OA at the (111) facets.^39^

This model, where *S_bound* OA ligands bind to Pb ions at the (111) facet and *W_Bound* OAH ligands bind to open sites at the (100) facet (Figure 4), enables us to quantify equilibrium constants for two free ⇋ *W_bound* and free ⇋ *S_bound* ligand exchange processes occurring in parallel. To calculate these equilibrium constants, we assume that *S_bound* and *W_bound* ligands form a monolayer on the QD surface. If a multilayer of ligands existed, we would expect van der Waals interactions to be present, but our control experiment with ODE confirms their absence. To further strengthen our monolayer assumption, we measured the QD particle size by using NMR diffusometry. The Stokes–Einstein equation (Equation S2 in Section S5) and the diffusion coefficient of the *S_bound* ligands yield a particle diameter (QD diameter +2 × ligand shell thickness) of 5.6 nm (Table S2 in Section S5). Notably, this size remains unchanged after titration with excess OAH, further indicating that multilayers do not form around the QD.

These combined measurements from NMR spectroscopy and diffusometry support our assumption of a single layer of ligands on the QD surface. Thus, we utilize the Langmuir adsorption model^45^ for the following equilibria, which operate in parallel.

3 free+UnoccupiedW(100)Sites⇌OccupiedW_boundSites

4 free+UnoccupiedS(111)Sites⇌OccupiedS_boundSites

We define the equilibrium constants of these ligand-binding reactions as

5 $$K_{\mathrm{W\_bound}}=\frac{\left[Occupied\;\mathrm{W\_bound}\;Sites\right]}{\left[\mathrm{Free}\;\mathrm{Ligand}\right]\left[Unoccupied\;W\left(100\right)\;Sites\right]}$$

6 $$K_{\mathrm{S\_bound}}=\frac{\left[Occupied\;\mathrm{S\_bound}\;Sites\right]}{\left[\mathrm{Free}\;\mathrm{Ligand}\right]\left[Unoccupied\;S\left(111\right)\;Sites\right]}$$

We describe *K_W_bound_* and *K_S_bound_* using concentrations expressed as the average ligand populations or surface sites per QD. This simplifies the molecular-level interpretation of concentrations. Under this framework, [Free Ligand] represents the average number of free OAH ligands per QD in solution. [Occupied *S_bound* Sites] corresponds to the average number of surface Pb sites per QD on (111) facets occupied by *S_bound* ligands, while [Occupied *W_*b*ound* Sites] represents the average number of surface Pb sites per QD on (100) facets occupied by *W_bound* ligands. We quantify both types of occupied surface sites based on bound ligand populations per QD, determined using ^1^H NMR spectroscopy and diffusometry (Table S4). [Unoccupied *W*(100) Sites] and [Unoccupied *S*(111) Sites] denote the total number of unoccupied Pb sites (per QD) on the (100) and (111) facets, respectively, which we determine using eqs 7 and 8.

7 [UnoccupiedW(100)Sites]=[AvailableW(100)Sites]−[OccupiedW_boundSite]

8 [UnoccupiedS(111)Sites]=[AvailableS(111)Sites]−[OccupiedS_boundSite]

For a QD with diameter 3.6 nm, we expect a total of 306 Pb surface atoms available for ligands to bind strongly or weakly. This number depends on the PbS QD’s size and crystal structure, with detailed assumptions and calculations described in Section S6.^9^ Distinguishing and quantifying [Available *W*(100) Sites] and [Available *S*(100) Sites] on the QD surface is challenging, but we can make predictions of these values based on the saturation of OA and OAH ligands on the QD surface.

Using the population fractions of ligands in three distinct states (Table 1) as a function of titrated OAH concentration, and using a ferrocene internal standard as a reference concentration, we determine the populations per QD of each of the three ligand states, [Free Ligand],[Occupied *S_*b*ound* Site], and [Occupied *W_*b*ound* Site], and present these in Table S5 and Figure 5.

**Figure 5:**
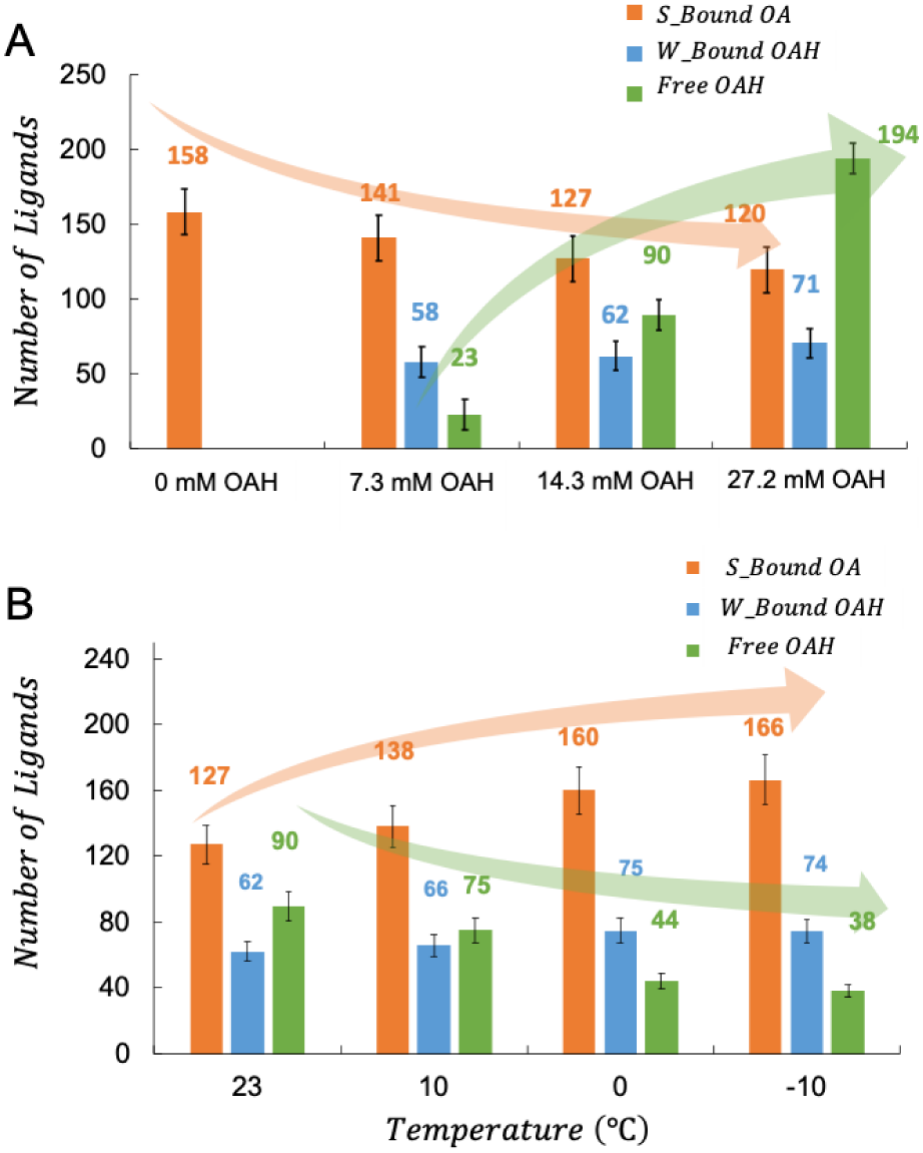
(A) Average number (populations) of ligands in different states per QD (surface-bound and in free solution) measured at 23 °C. Ligand populations in different states change as the OAH titration concentration increases from 7.3 to 27.2 mM. (B) Ligand population changes for 150 μM OA-capped PbS QDs with 14.3 mM of OAH titrated into the solution in toluene-d_8_ from 23 °C to −10 °C (on cooling). Increasing the acidity (OAH) of the solution reduces OA binding on the QD surface, and lowering the temperature improves ligand binding on the QD surface.

When the concentration of excess OAH is increased from 7.3 mM to 27.2 mM, the number of *S_bound* ligands per QD gradually decreases (Figure 5A). One potential explanation for this decrease is that the increased concentration of acidic protons from free OAH weakens the OA–Pb bond by protonating the surface-bound OA, leading to a reduction in the *S_bound* ligand population (and the observed NMR signal). Assessment of this hypothesis requires further investigation.

Despite the decrease in *S_bound* ligand population (158 to 120, Figure 5A), the total number of surface-bound ligands per QD (*S_bound* + *W_bound* = 193 ± 6) remains unchanged across samples titrated with 7.3 mM, 14.3 mM, and 27.2 mM excess OAH at 25 °C. This could be attributed to the higher enthalpy of free OAH ligands in a nonpolar solvent like toluene, which favors ligand binding to the QD surface to achieve lower enthalpy. These findings provide insights into the dynamic equilibrium of ligand binding on QD surfaces, as well as the interplay between solvent effects and surface interactions.

Since both enthalpic and entropic effects play a crucial role in ligand-QD interactions, we measured ligand populations as a function of temperature (Figure 5B). For OA-capped PbS QDs titrated with 14.3 mM of excess OAH, decreasing the temperature from 23 °C to −10 °C resulted in an increase in the total population of surface-bound ligands and a corresponding decrease in free OAH. In the temperature range of measurement, both enthalpy (Δ*H*) and entropy (Δ*S*) are expected to exhibit minimal variation, although the ligand packing increases modestly from 4.7 ± 1 ligands/nm^2^ at 23 °C to 5.9 ± 1 ligands/nm^2^ at −10 °C. Therefore, the primary factor driving the decrease in Gibbs free energy (Δ*G*) is the entropic term (*T*Δ*S*), with temperature (*T*) being the dominant variable that changes significantly in this regime. This trend aligns with thermodynamic principles: at higher temperatures, entropy favors free ligands with greater degrees of freedom, while at lower temperatures, reduced entropic contributions to Gibbs free energy promote exothermic ligand binding to the QD surface.

Temperature-dependent ligand population studies also revealed the near saturation of *S_bound* (190 OA/QD) and *W_bound* (77 OAH/QD) ligands on the QD surface at 27.2 mM OAH (Table S5). As described above, this saturation represents near-complete occupation of Pb atoms on the (100) and (111) facets by *W_bound* and *S_bound* ligands, respectively. Based on the ligand saturation at a maximum OAH concentration of 27.2 mM and the total available Pb sites per QD extracted from the VESTA model, we estimate that the total Available *S*(111) Sites = 210 and the total Available *W*(100) Sites = 100 when accounting for Pb sites not completely bound by OA and OAH ligands. Assuming these values enables determination of the equilibrium constants (*K_W_bound_* and *K_S_bound_*) for each sample as a function of temperature (Table 2).

**Table 2:** Exchange Equilibrium Constants for *S_Bound* OA Ligand Binding (*K_S_bound_*, eq 6) and *W_Bound* OAH Ligand Binding (*K_W_bound_*, eq 5) on 3.6 nm PbS QDs

	QD + 7.3 mM OAH		QD + 14.3 mM OAH		QD + 27.2 mM OAH	
Temperature (°C)	*K_S_bound_*	*K_W_bound_*	*K_S_bound_*	*K_W_bound_*	*K_S_bound_*	*K_W_bound_*
23	0.088 ± 0.02	0.06 ± 0.027	0.017 ± 0.003	0.018 ± 0.005	0.007 ± 0.001	0.013 ± 0.003
10	0.161 ± 0.05	0.041 ± 0.018	0.026 ± 0.005	0.026 ± 0.007	0.019 ± 0.003	0.021 ± 0.004
0	0.291 ± 0.09	0.024 ± 0.1	0.073 ± 0.015	0.068 ± 0.017	0.015 ± 0.003	0.021 ± 0.004
–10	0.585 ± 0.17	0.076 ± 0.034	0.097 ± 0.02	0.077 ± 0.02	0.078 ± 0.012	0.024 ± 0.005

From the quantified *K_W_bound_* and *K_S_bound_* at various temperatures, we can also determine thermodynamic parameters (Δ*H*, Δ*S*, and Δ*G*) using Van’t Hoff plots (Figure S11). Thermodynamic analysis of ligand binding to the QD surface (Section S9) highlights the intricate balance of enthalpic and entropic contributions governing surface interactions. As mentioned above, our results show exothermic ligand binding, with stronger OA binding to (111) facets (Δ*H_S_bound_* = −38.7 ± 3 kJ/mol) compared to OAH binding to (100) facets (Δ*H_W_bound_* = −21.4 ± 13.6 kJ/mol).

Interestingly, binding spontaneity decreases with increasing OAH concentration for both *S_bound* and *W_bound* ligands. This trend might be attributed to (1) reduced availability of surface binding sites and (2) protonation-induced weakening of existing *S_bound* ligands on the QD surface. While enthalpy values align with theoretical predictions,^39^ the (slightly) positive Gibbs free energy (Δ*G*) values indicate nonspontaneous binding and present an intriguing anomaly. This unexpected behavior could suggest additional factors influencing ligand-QD interactions that are not fully accounted for in our current model and may involve, for instance, ligand-ligand interactions in the free solution state or surface site binding cooperativity. Future work focused on systematic variations in experimental conditions, including solvent composition and ligand identity, will aid in refining our understanding of this and related systems.

### Kinetics of Fast Exchange between Free Ligands and W-Bound Ligands

Chemical exchange commonly appears in NMR spectra, often evident in the broad signals arising in the ^1^H spectra of hydroxyls and amines.^46–52^ Such exchange is observed in our samples, where we detect a broad exchanging signal (Figure 2, blue trace, i.e., the “intermediate” signal) near 5.7 ppm. As introduced above, this signal arises from fast exchange, compared to the NMR spectroscopy time scale of measurement (see also Section S2), between *W_bound* and free OAH ligands. On this time scale (3.9 ms at 23 °C), we observe the simultaneous occurrence of both forward binding and backward dissociation of an OAH ligand to an unoccupied (100) Pb site, with rate constants *k_W_Bind_* and *k*_dissoc_, respectively, as given by eq 9.

9 $$\mathrm{Free}\;\mathrm{OAH}+\mathrm{Unoccupied}\;W\left(100\right)\;\mathrm{Sites}\overset{k_{W-Bind}}{\underset{k_{dissoc}}{⇌}}\mathrm{W\_Bound OAH}$$

The line shape and chemical shift of this exchanging peak (intermediate peak) depend on the exchange rate of the probed nuclei and the population fraction of the ligands in the two states, as shown in Figure S13 in Section S10. From^1^H NMR spectroscopy (Figure S6) and diffusometry measurements (Table 1), we can confirm that the OAH ligand exchanges rapidly between free and *W_bound* states for all three of our OAH-titrated samples (7.3 mM, 14.3 mM, and 27.2 mM). To quantify the rate of such an exchange process, Rogers and Woodbrey^46^ developed a generalized line shape function *S*_ex_(*v*) for a two-site exchange model as described below (eqs 10–16). Gutowsky and Holm initially applied this model to study amide bond rotation kinetics.^50^

10 $$S_{ex}\left(\nu \right)=C_{0}\times \left(\frac{P\left[1+\tau_{ex}\left(\frac{p_{solv}}{T_{2,\mathrm{W\_bound}}}+\frac{p_{\mathrm{W\_bound}}}{T_{2,\mathrm{free}}}\right)\right]+QR}{{\left(P\right)}^{2}+{\left(R\right)}^{2}}\right)$$

11 $$P=\tau_{ex}\times \left(\frac{1}{T_{2,\mathrm{W\_bound}}\times T_{2,free}}-4\pi^{2}\Delta \nu^{2}+\pi^{2}\delta \nu^{2}\right)+\frac{p_{\mathrm{W\_bound}}}{T_{2,\mathrm{W\_bound}}}+\frac{p_{free}}{T_{2,free}}$$

$$Q=\tau_{ex}\times \left(2\pi \Delta \nu -\pi \delta \nu (p_{\mathrm{W\_bound}}-p_{free})\right)$$ 12

13 $$R=2\pi \Delta \nu \left[1+\tau_{ex}\left(\frac{1}{T_{2,\mathrm{W\_bound}}}+\frac{1}{T_{2,free}}\right)\right]+\pi \delta \nu \tau_{ex}\left(\frac{1}{T_{2,\mathrm{W\_bound}}}-\frac{1}{T_{2free}}\right)+\pi \delta \nu \left(p_{\mathrm{W\_bound}}-p_{free}\right)$$

14 $$\delta \nu =\left|\nu_{\mathrm{W\_bound}}-\nu_{free}\right|$$

15 $$\Delta \nu =\left(\frac{\nu_{free}+\nu_{\mathrm{W\_bound}}}{2}\right)-\nu$$

16 $$\tau_{ex}=\frac{p_{\mathrm{W\_bound}}}{k_{\mathrm{W\_Bind}}}=\frac{p_{free}}{k_{dissoc}}$$

The above NMR signal line shape function *S*_ex_(*v*) depends on the exchange lifetime τ_ex_ (eq 16), which represents the overall exchange time for weak ligand binding and dissociation. This exchange time is the reciprocal of the overall exchange rate constant *k*_ex_ = *k_W_Bind_* + *k*_dissoc_ = τ*_ex_* ^–1^. Despite its complexity, the line shape function (eq 10) requires only six known parameters to fit the experimental data and determine τ_ex_. These parameters are the spin–spin relaxation times (*T*_2,free_, *T*_2,_ *_W_bound_*) and the chemical shifts (*ν*_free_, *ν_W_bound_*) obtained for the free and *W_bound* ligand states, as well as the instantaneous population fractions $\left(p_{free}=\frac{f_{free}}{f_{ex}}\;\mathrm{and}\;p_{\mathrm{W\_Bound}}=\frac{f_{\mathrm{W\_Bound}}}{f_{ex}}\right)$ . In this function, δ*ν* represents the chemical shift difference between the two exchanging sites, and Δ*ν* denotes the midpoint frequency between the *free* and *W_bound* ligand signals as shown in eqs 14 and 15. *C*_0_ is an intensity scaling factor determined by fitting the experimental data with the line shape function.

We determine the spin–spin relaxation times for the nonexchanging free ligand (*T*_2,free_) and *W_bound* ligand (*T*_2,_ *_W_bound_*) protons as follows. Since we cannot separately measure the *W_bound* ligands, but the alkenyl protons of *S_bound* and *W_bound* ligands have essentially identical chemical (magnetic) environments, we can assume that *T*_2,_ *_W_bound_* = *T*_2,_ *_S_bound_* and then measure *T*_2,_ *_S_bound_* on a sample containing only *S_bound* ligands without excess OAH. To measure *T*_2_ values with the best accuracy when J-couplings (spin–spin splittings) are present, we used a modified CPMG pulse sequence (see Section S11).^53^ We determine the population fractions *p*_free_ and *p_W_bound_* from NMR diffusometry measurements using eq 1 Finally, we determine *ν*_free_ from a sample containing only free OAH in toluene-d_8_ and then determine *ν_W_bound_* using eq 2.

We then determine the exchange lifetime τ_ex_ by least-squares fitting the separated exchange NMR spectroscopy signal with eq 10 using code written in Python. Similarly, we fit the *S_bound* signal with Equation S8. Figure S16 displays the fits (dashed line) of the acquired 1D spectra (solid line) of a sample containing OA-capped PbS QDs titrated with 14.3 mM of OA measured at 23 °C. The excellent agreement of the spectral fitting confirms that the obtained τ_ex_ from eq 10 accurately represents the expected exchange process between *W_bound* and free ligands. Table 3 lists the parameters used to determine τ_ex_, which in turn gives *k_W_Bind_* for weak ligand binding. The exchange rate decreases with increasing OAH concentration, which we attribute to the saturation of *W*(100) sites on the QD surface and the associated ligand chain crowding. Figure 6 below summarizes ligand behavior in the solution, along with the observed changes in the energetics when we increase the OAH titration concentration.

**Figure 6:**
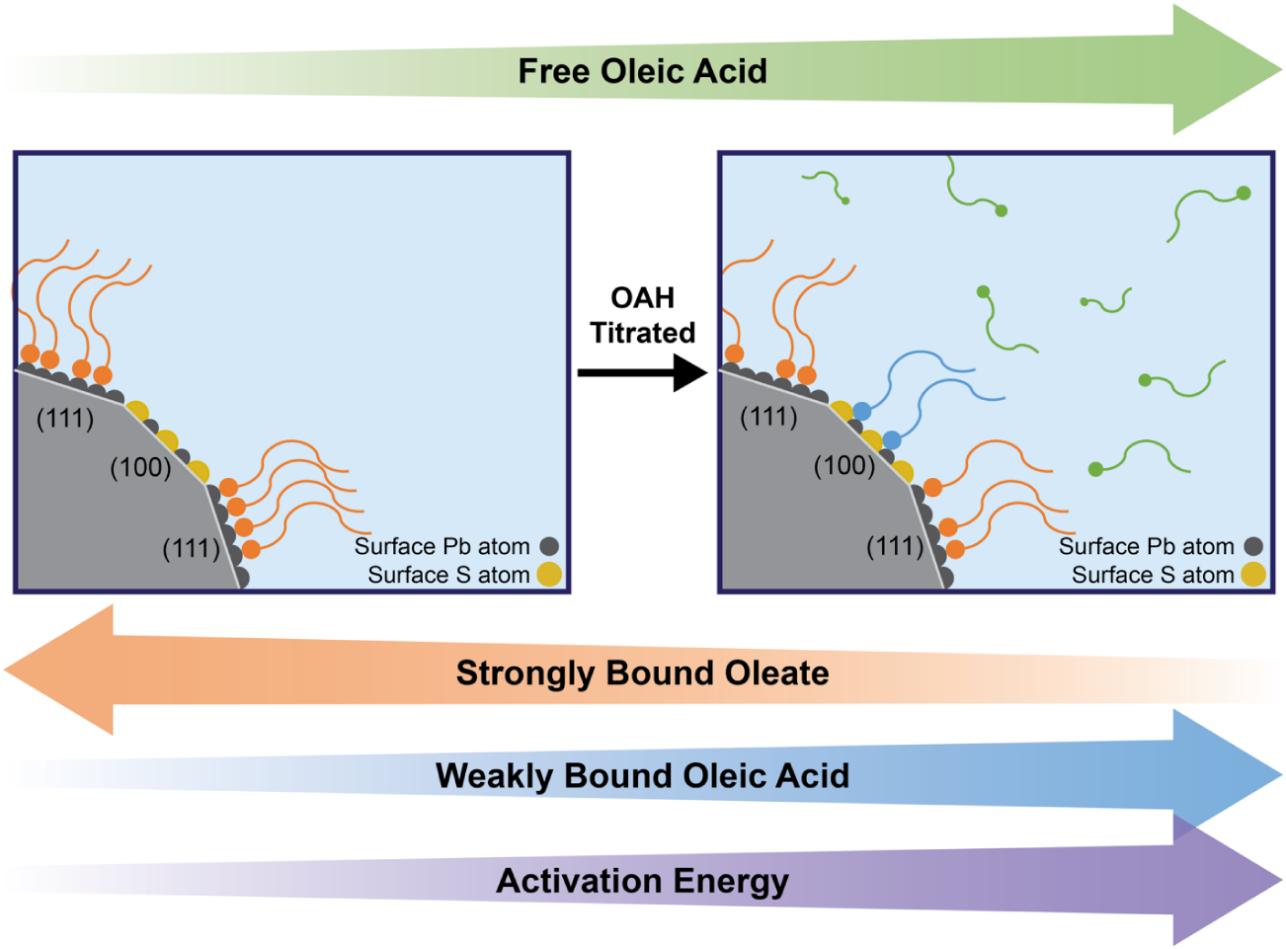
Changes in population fractions of the ligand in different states (*S_bound*, *W_bound*, and free) and activation energy for weak ligand binding to the QD surface with an increase in the OAH titration concentration.

**Table 3:** Weak Ligand Binding Rate (*k_W_Bind_*) Dependence on Concentration of Titrated OAH^a^

Sample	*p_W_bound_* (%)	*p*_free_ (%)	*ν*_free_ (ppm)	*ν*_*W_bound*_ (ppm)	*T*_2,free (s)_	*T*_2,*W_bound* (*s*)_	*τ*_ex_ (ms)	*k_W_Bind_* (s^–1^)
QD + 7.3 mM OAH	29	71	5.58	5.74	0.78	0.053	0.89 ± 0.1	490 ± 50
QD + 14.3 mM OAH	59	41	5.58	5.76	0.78	0.053	0.91 ± 0.1	450 ± 50
QD + 27.2 mM OAH	73	27	5.58	5.76	0.78	0.053	1.5 ± 0.1	300 ± 50

a We determine the weak ligand binding rate *k_W_Bind_* from the exchange lifetime τ_ex_ and eq 16. *k_W_Bind_* did not change (within error) when increasing the OAH titration concentration from 7.3 mM to 14.3 mM because the occupied W(100) sites on the QD surface are not saturated. When further increasing the OAH titration concentration to 27.2 mM, the occupied *W*(100) sites become saturated, resulting in a decrease in *k_W_Bind_*.

We further measure the activation energies (*E_a,W_Bind_*) of the OAH weak binding process by analyzing the temperature dependence of *k_W_Bind_*. For simplicity, we assume the *k_W_Bind_* temperature dependence follows the Arrhenius equation:

17 $$k_{\mathrm{W\_Bind}}=k_{\infty ,\mathrm{W\_Bind}}e^{-\frac{E_{a,\mathrm{W\_Bind}}}{RT}}$$

where *R* is the gas constant and *T* is the solution temperature. *k*_∞,_ *_W_Bind_* denotes the weak ligand binding rate at infinite temperature, also known as the “barrierless reaction rate.” This empirical relationship enables determination of *E_a,*W_Bind*_*.

Table 4 presents *E_a,*W_Bind*_* for samples containing varying OAH titration concentrations. At low OAH concentrations (7.3 mM), *E_a,W_Bind_* = 5.2 ± 5 kJ/mol, which increases to 15–20 ± 5 kJ/mol when the OAH concentration is ≥14.3 mM. This apparent rise in *E_a,W_Bind_* agrees with the idea of a chain crowding effect that elevates the energy barrier for ligand binding on the surface.^54,55^ Lastly, based on the measured values for *E_a,W_Bind_* and the lower bound for the exchange rate between *S_bound* and free states (200 ms), we discuss a coarse estimation of *E_a,S_Bind_* in Section S13. These findings contribute to our understanding of the complex interplay between the ligands and the QD surface.

**Table 4:** Temperature-Dependent Ligand Binding Rates (*k_W_Bind_*) and Activation Energies of *W_Bound* OAH Ligands on the (100) Facet of a 3.6 nm PbS QD

	OA-capped PbS QD + 7.3 mM OAH (50 equiv)		OA-capped PbS QD + 14.3 mM OAH (100 equiv)		OA-capped PbS QD + 27.2 mM OAH (200 equiv)	
Temperature (°C)	*k_W_Bind_* (s^–1^)	*E_a,W_Bind_* (kJ/mol)	*k_W_Bound_* (s^–1^)	*E_a,W_Bind_* (kJ/mol)	*k_W_Bind_* (s^–1^)	*E_a,W_Bind_* (kJ/mol)
23	490 ± 50	5.2 ± 5	450 ± 50	20.5 ± 5	300 ± 50	15.5 ± 5
10	290 ± 50		390 ± 50		280 ± 50	
0	315 ± 50		170 ± 50		240 ± 50	
–10	360 ± 50		190 ± 50		130 ± 50	

## Conclusions

This study significantly advances the understanding of oleic acid (OAH) ligand binding with PbS quantum dots (QDs) by updating the conventional two-state ligand model (bound and free) to a more comprehensive three-state model: strongly bound (*S_bound*), weakly bound (*W_bound*), and free ligands. We validate this model using nondestructive ^1^H NMR spectroscopy and diffusometry measurements in solution, which enables us to quantify ligand population fractions in each state and track their variations as a function of temperature and of free OAH concentration.

This study also enables us to determine, in solution, the number of OA sites (X-type) and OAH sites (L-type) on PbS QDs, which is generally intractable using traditional techniques. Furthermore, applying the Langmuir adsorption model to our data provides thermodynamic parameters (Δ*H* and Δ*S*) for both strong and weak ligand binding to the QD surface.

By leveraging NMR’s ability to probe molecular dynamics, we additionally investigate ligand exchange kinetics, revealing fast OAH exchange between free and *W_bound* ligand states. Using a temperature-dependent “dynamic NMR" line shape analysis, we determine exchange rates and activation energies between free and *W_bound* ligands, offering key insights into the energetics of ligand-QD interactions. Our findings highlight NMR spectroscopy’s power in studying complex molecular systems, enabling model validation and dynamic/kinetic analysis.

Future studies will explore ligand-binding energetics when the titrated ligand differs from the native ligand. This work lays the foundation for further investigations in computational modeling, QD structure–property relationships, *in situ* exchange studies, and novel ligand design. Beyond advancing our fundamental understanding of QD surface chemistry, our study provides a powerful framework for the rational design and optimization of QD-based materials and devices. More specifically, these results are a step toward achieving fine control over nanoparticle reactivity by selecting appropriate stabilizing ligands and tailoring their dynamics. Such control over surface chemistry represents a powerful strategy for optimizing QD performance in applications such as optoelectronics, catalysis, and quantum-scale sensing technologies.

## Methods

### General Solvent and Starting Material Considerations

Solvents, including toluene, acetone, and pentane, which are used for the purification of QDs and for UV–visible–NIR absorbance measurements, were purchased from VWR. Toluene-d_8_ was purchased from Cambridge Isotope Laboratories. Lead­(II) oxide (99.999%), oleic acid (OAH) (90%), 1-octadecene (ODE) (90%), and ferrocene (98%) were purchased from Sigma-Aldrich and used as received. Bis­(trimethylsilyl) sulfide ((TMS)_2_S) (≥98%) was purchased from Sigma-Aldrich and stored under N_2_.

### Synthesis and Purification of PbS QDs

Standard Schlenk line techniques were used to maintain inert conditions during the PbS QD synthesis. A modified version of the procedure established by Hines and Scholes was followed.^3,17^ Lead­(II) oxide (0.91 g, 4 mmol), OAH (2.54 mL, 8 mmol), and ODE (35.5 mL) were combined in a 100 mL three-neck round-bottom flask and stirred under a vacuum at 100 °C for 2.5 h. The mixture was then heated to 120 °C under a N_2_ atmosphere to yield a clear, colorless solution indicative of Pb­(OA)_2_ formation.

Next, (TMS)_2_S (0.42 mL, 2 mmol) and ODE (5 mL) were combined in a 25 mL pear-shaped flask under an inert atmosphere. The (TMS)_2_S solution was swiftly injected into the Pb­(OA)_2_ solution at 125 °C. The reaction proceeded at 123 °C for 2.5 min, turning dark brown as it continued. The reaction flask was removed from the heating mantle, and the QD solution was quenched by first being submerged in a room-temperature oil bath for 3 min followed by an ice bath for 4 min. Three-milliliter aliquots of the reaction mixture were transferred to centrifuge tubes, diluted with 1 mL of toluene, precipitated with 9 mL of acetone, and centrifuged at 9000 rpm for 10 min.

After decanting the supernatant, the QDs were resuspended in 4 mL of pentane, precipitated by adding 4 mL of methanol and 4 mL of acetone, and centrifuged at 10,600 rpm for 10 min. Four additional precipitation–centrifugation cycles were carried out, alternating between 2 mL of pentane or toluene and 8 mL of acetone for the first two steps, and 6 mL of acetone for the last two. The PbS QDs were isolated from pentane by evaporation, yielding 0.9967 g of QDs.

### ^1^H NMR Spectroscopy for Titrations

Samples for ^1^H NMR spectroscopy were prepared by determining the concentration of a PbS QD stock solution in toluene-d_8_ after measuring the absorbance of 10 μL of the PbS QD stock solution in 3.0 mL of toluene. A predetermined volume of the stock solution was diluted with toluene-d_8_ to obtain a concentration of 150 mM QDs in 600 μL. An internal standard solution of ferrocene was prepared by dissolving a precise amount (ca. 10 mg in 1.0 mL of toluene-d_8_), and 10 μL of this solution was added to 590 μL of a diluted QD stock in each NMR spectroscopy tube to reach the desired concentration and volume. A precise solution of OAH in toluene-d_8_ was then prepared, and additions of 50 (7.3 mM), 100 (14.3 mM), and 200 (27.2 mM) equivalence per QD were added to the NMR spectroscopy tubes. For the addition of 1-octadecene (ODE), a stock solution was also prepared in toluene-d_8_, and 100 equiv per QD (14.3 mM) was added to the NMR spectroscopy tubes with the QD solution. All samples were prepared under inert conditions in a N_2_ atmosphere glovebox. Four 1 mm capillaries were added to each NMR spectroscopy tube to prevent artifacts in diffusion measurements due to thermal convection. All samples were flame-sealed to preserve the inert atmosphere and ensure the longevity of the sample.

All ^1^H NMR spectroscopy measurements were made on a 600 MHz Bruker Avance III spectrometer equipped with a 5 mm TCI Prodigy cryoprobe (^1^H inner coil). Measurements were acquired over a temperature range of −10 to +23 °C. For quantitative accuracy, 8 scans with a 15° pulse angle and a very long relaxation delay of 100 s between each scan were used. This is because ferrocene in inert conditions has a very long spin–lattice relaxation time (*T*_1_). This ensured that >99.9% of the signal was recovered for each scan, enabling accurate quantification. More details on the choice of parameters are explained in Section S7. All spectra were processed using MNova, and the multipeak fitting function was used to integrate the alkenyl peaks in ^1^H NMR to determine the concentrations of bound and unbound ligands.

### UV–Visible–NIR Absorbance Measurements

UV–visible–NIR absorbance spectra of as-synthesized PbS QDs were collected on an Agilent Cary 60 spectrometer in toluene. Concentrations of PbS QDs were calculated using the absorbance value at 400 nm and the ε_400_ value determined from established methods by Moreels and coworkers.^11^

### Transmission Electron Microscopy (TEM)

Samples for TEM analysis were prepared by drop-casting diluted PbS QD solutions in pentane onto 400-mesh lacey carbon grids (Ted Pella, Inc.). All samples were dried overnight under a vacuum at room temperature. TEM analysis was conducted using a Thermo Fisher Talos F200X at an accelerating voltage of 200 kV. The imaging was done in TEM mode. Images were analyzed by using the software ImageJ.

### ^1^H Pulsed-Field-Gradient (PFG) NMR Diffusometry

All NMR diffusometry experiments were performed using a 400 MHz Bruker Avance III WB NMR spectrometer equipped with an a^1^H 5 mm coil coupled to a Diff50 single-axis (*z*-axis) gradient coil. We employed the pulsed-field-gradient stimulated echo (PGSTE) sequence to measure the diffusion coefficients of ligand species present in the solutions. In this experiment, the NMR signal intensity *I* for each distinct peak observed in ^1^H NMR spectroscopy was measured as a function of gradient strength (*g*). This acquired signal intensity attenuation dependence was fit using the Stejskal–Tanner equation:^56–58^

18 $$I=I_{0}e^{-\gamma^{2}g^{2}\delta^{2}\left(\Delta -\frac{\delta }{3}\right)D}=I_{0}e^{-bD}$$

where *I*_0_ is the signal amplitude at *g* = 0, γ is the gyromagnetic ratio, δ is the effective gradient pulse length, Δ is the diffusion time between gradient pulses, and *D* is the self-diffusion coefficient. The “*b*” factor, representing all the known NMR-specific parameters and useful for quantifying diffusion behaviors, is given by *b* = γ^2^ *g* ^2^δ^2^(Δ – δ/3). The sequence was used with a 90° RF pulse length of 4.5 μs and an effective gradient pulse length δ = 1 ms. The diffusion time Δ was set to 25 ms. The maximum gradient strength (*g*_max_) was varied from 60 to 400 G·cm^–1^ to achieve more than 95% signal attenuation in 8 to 32 steps. Faster diffusing species require smaller *g*_max_ and slower diffusing species require larger *g*_max_. A sufficient signal-to-noise ratio (SNR) was achieved with 64–256 scans and acquisition times of 0.2–2 s depending on the chemical species probed (longer acquisition times for solvent molecules). Figure S18 in compares the ^1^H NMR spectra for the 1D pulse-acquire and PGSTE first slice for OA-capped PbS QDs titrated with 7.3 mM free OAH. Spin–lattice relaxation time (*T*_1_) measurements using the inversion–recovery sequence and the same RF pulse lengths as above yield *T*_1_ values for *S_bound* and free ligand of 0.85 s and for ferrocene of 30 s. A modified CPMG pulse sequence was used (see Section S11 for more details) to compensate for J-modulation effects and obtain accurate spin–spin relaxation times *T*_2_ for ligands and toluene molecules.^53^ *T*_2_ values for *S_bound* and free ligands are listed in Table S7. The choice of parameters used for PGSTE experiments did not produce any significant differences in signal intensity weighting (see Figure S18) due to *T*_1_ and *T*_2_ spin relaxation variations.

A relaxation delay of 2 s was used between each scan for experiments to probe ligand signals (which required higher gradients), and a relaxation delay of 6 s was used for experiments to probe the solvent toluene-d_8_ signal (lower gradients) to achieve sufficient SNR and measure their respective diffusion coefficients. Two Hz line broadening was applied during data processing to reduce excess acquisition noise. All NMR experiments were performed at temperatures ranging from −10 ± 1 °C to 23 ± 1 °C to observe the changes in thermodynamics and kinetics of ligand interactions at the QD surface.

## Supplementary Material



## Notes

NMR spectra of pure OAH, UV–vis of QDs in toluene-d_8_, TEM micrograph of the QDs, size distribution of QDs, NMR time scale for 1D NMR and NMR diffusometry measurements, ^1^H NMR spectrum and diffusion of 1-octadecene with and without PbS QDs, PbS QD and ligand shell size determination using NMR diffusometry, quantification of Pb atoms on the surface of 3.6 nm OA-capped PbS QDs, ligand population quantification in different states, thermodynamic parameter determination from the exchange equilibrium constants, exchange kinetics from dynamic NMR spectroscopy, spin–spin relaxation time measurements from modified CPMG, fast-exchanging ligand signal fitting using Python and line shape function, activation energy for slow exchange processes, spectrum comparison of 1D pulse and PGSTE 1st slice, acidic proton variation with OAH titration concentration and temperature (PDF)
