# Supplementary material for: Ligand Exchange and Binding at the Surface of PbS Quantum Dots Quantified Using Multimodal Magnetic Resonance
Source: ACS Nano. 2025 Jul 22;19(30):27246–58. doi: 10.1021/acsnano.5c03943 (PMC12333426; doi:10.1021/acsnano.5c03943)
Supplement: Supplementary file 1 [file nn5c03943_si_001.pdf]

Supporting Information

Ligand Exchange and Binding at the Surface of PbS Quantum Dots Quantified Using Multimodal Magnetic Resonance

*Veera Venkata Shravan Uppala[1], Christian Y. Dones Lassalle,[2] Jennica E. Kelm[2], Andrew M. Camp,[2] Marc A. ter Horst,[2] Alan R. Esker,[1] Jillian L. Dempsey, [*2] and Louis A. Madsen[1*]*

[1]Department of Chemistry and Macromolecules Innovation Institute, Virginia Polytechnic Institute and State University, Blacksburg, Virginia 24061, United States

[2]Department of Chemistry, University of North Carolina, Chapel Hill, North Carolina 27599-3290, United States

Louis A. Madsen: lmadsen@vt.edu

Jillian L. Dempsey: dempseyj@email.unc.edu

1. ^{1}H NMR Spectrum of Free Oleic acid in Toluene-d_8 and Additional Characterization of PbS QDs

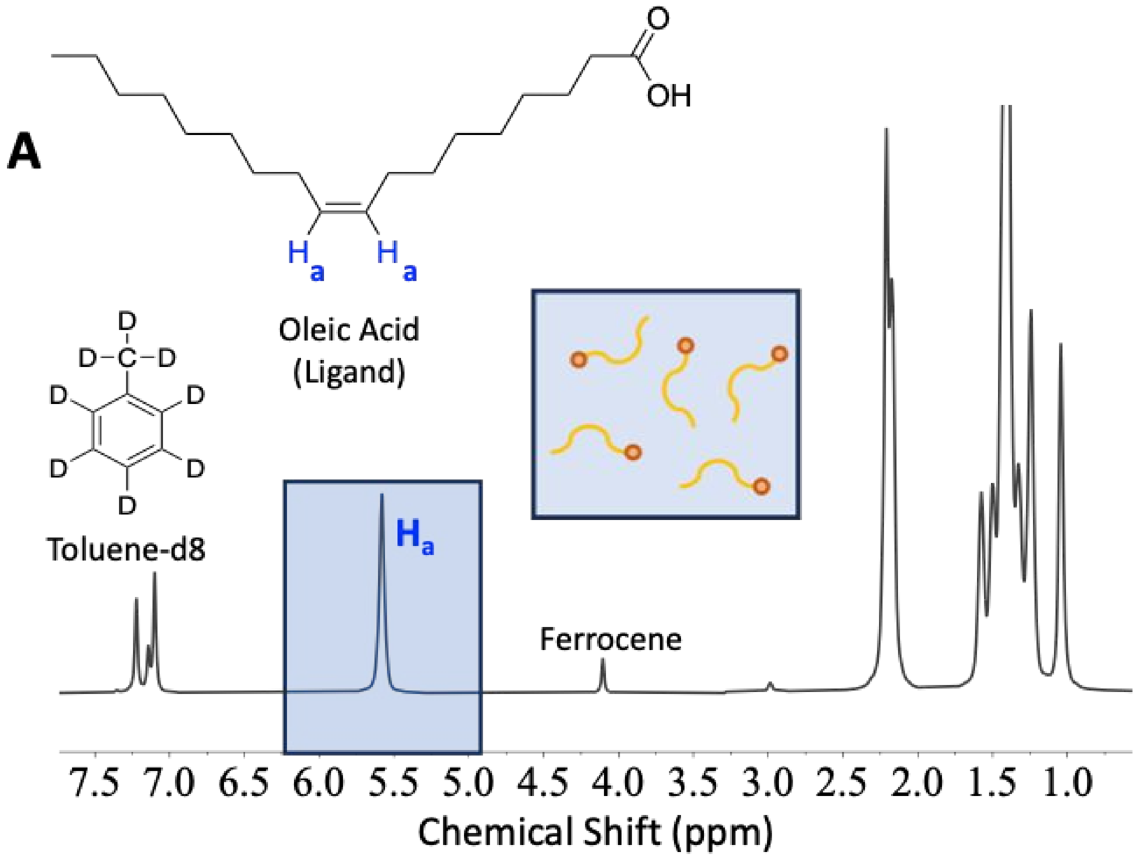

Figure S1: ^{1}H NMR spectrum of free oleic acid (OAH) in toluene-d_8 with a ferrocene internal standard of known concentration (see the Methods section in the manuscript for details). The alkenyl proton signal (H_a) at 5.6 ppm is well resolved from the remaining alkyl proton signals below 2.4 ppm, toluene peaks near 7 ppm, and internal standard ferrocene near 4.1 ppm. Thus, H_a is probed to quantify ligand dynamics and population fractions.

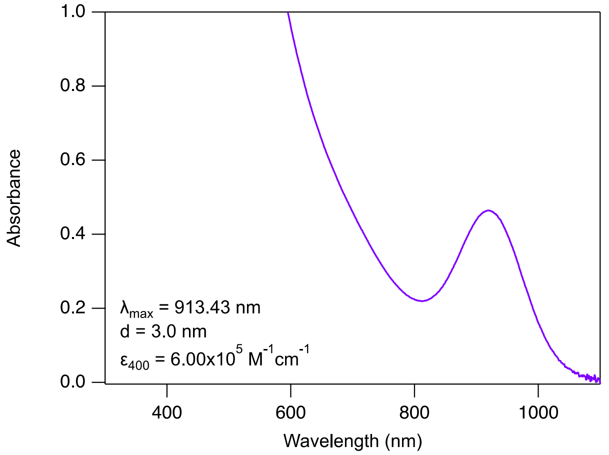

Figure S2: UV-Vis-NIR absorbance spectrum of as-synthesized PbS QDs in toluene. The QD diameter (3.0 nm) was calculated using the empirical sizing curve derived by Moreels et al.[1]

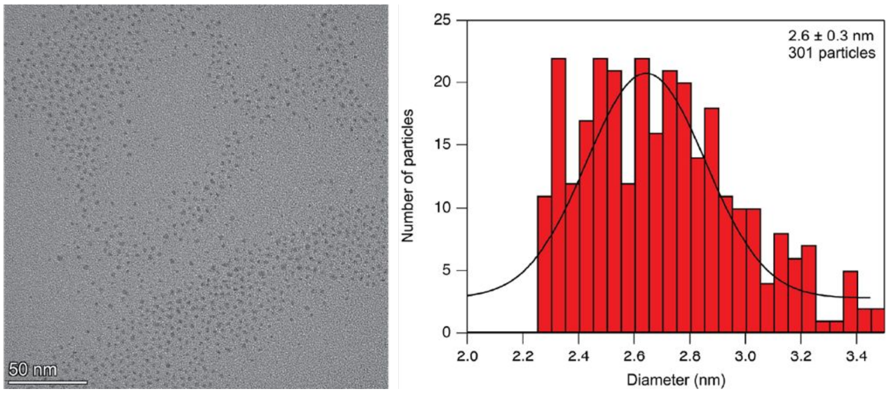

Figure S3: TEM image of as-synthesized PbS QDs (left). Histogram of 301 particles (right). Fitting the histogram to a Gaussian distribution results in an average QD diameter of 2.6 ± 0.3 nm.

2. Explanation of Distinct Timescales for 1D NMR and NMR Diffusometry Measurements

NMR spectroscopy, beyond its molecular structure determination capability, can also probe dynamics across multiple time and length scales. The timescale of a particular NMR measurement depends on the *experiment type* and the *states of the nuclei* observed. In a simple 1D spectroscopy NMR measurement involving exchanging nuclear environments, the timescale depends on the chemical shift difference ($\delta\nu$) of the two molecular states of the nucleus observed. In our study, we can only observe the dynamics of oleic acid (OAH) and oleate (OA) ligands bound to the QD

surface via the strongly bound signal (ν_{S_Bound}) and the signal for the free oleic acid (ν_{free}) in the solvent. Hence the NMR timescale (τ) for a 1D NMR spectroscopy measurement is given by **Equation S1**:

$$\frac{1}{timescale\ (\tau)} = \frac{\pi \cdot \delta\nu}{\sqrt{2}} \qquad \text{S1}$$

where $\delta\nu = \nu_{S\_Bound} - \nu_{free}$ is the chemical shift difference between the oleic acid ligand in bound and free states, as shown in **Figure S4** below. The NMR timescale for a 1D spectrum of the OA-capped QD system probed in this work measured on a 600 MHz instrument is $\tau = 3.9\ ms$. However, in an NMR diffusometry experiment, the relevant timescale τ depends on the user-defined experimental parameter known as the "diffusion time" (Δ) (details of Δ are in the experimental section of the manuscript). For almost all of our NMR diffusometry measurements, we set $\Delta = 25\ ms$ to quantify ligand diffusion coefficients. However, for some control experiments, we increased $\Delta = 200\ ms$ to observe any potential changes to the NMR spectrum of the alkenyl proton signal. We did not observe any changes in the signal profile with varying Δ. This study thus provides at least a qualitative understanding of the exchange time for OA (W_bound) ligand binding/unbinding, which must be substantially larger than $\tau = 200\ ms$.

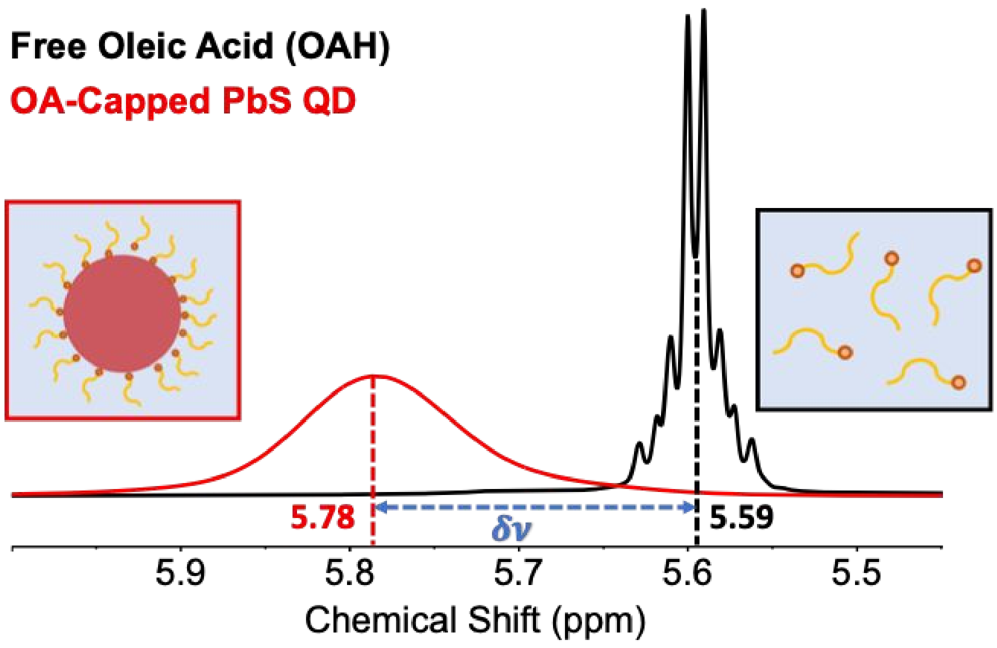

Figure S4: ^{1}H NMR spectra of 150 μM 3.6 nm OA-capped PbS QDs and free oleic acid (OAH) measured on a 600 MHz instrument. The chemical shift difference ($\delta\nu$) between the two states tells us about the timescale of the measurement.

3. ^{1}H NMR Spectrum of 1-octadecene and Diffusion Studies With and Without PbS QDs

The diffusion coefficients of free 1-octadecene (ODE) and ODE in an OA-capped PbS QD solution are both 12×10^{-10} $m^2\cdot s^{-1}$, which is nearly an order of magnitude higher than that of the QDs and the bound oleate (OA) ligands (1.3×10^{-10} $m^2\cdot s^{-1}$), as shown in **Figure S5**. If ODE were to interact with QDs, NMR diffusometry would reveal either two distinct diffusion coefficients (corresponding to free and bound species) or a single, slower, weighted-average diffusion coefficient if ODE exchanged rapidly on the diffusion timescale.

However, we observed identical diffusion coefficients for ODE in both the control and the QD-containing solutions, indicating that ODE diffuses freely and does not bind to the QD surface through van der Waals interactions. Moreover, the measured diffusive displacement of ODE is 7.8 μm, which is four orders of magnitude larger than its molecular size and three orders of magnitude

larger than the QD size. This large displacement means that NMR diffusometry in general cannot detect any differences in diffusion coefficients based on signal contributions from different sites on the ODE molecule. These NMR diffusometry studies from control experiments also confirm that W_Bound oleic acid (OAH) ligands interact with the QD surface not through van der Waals forces between alkenyl chains, but rather though coordination involving the acidic head group – a functional group absent in ODE.

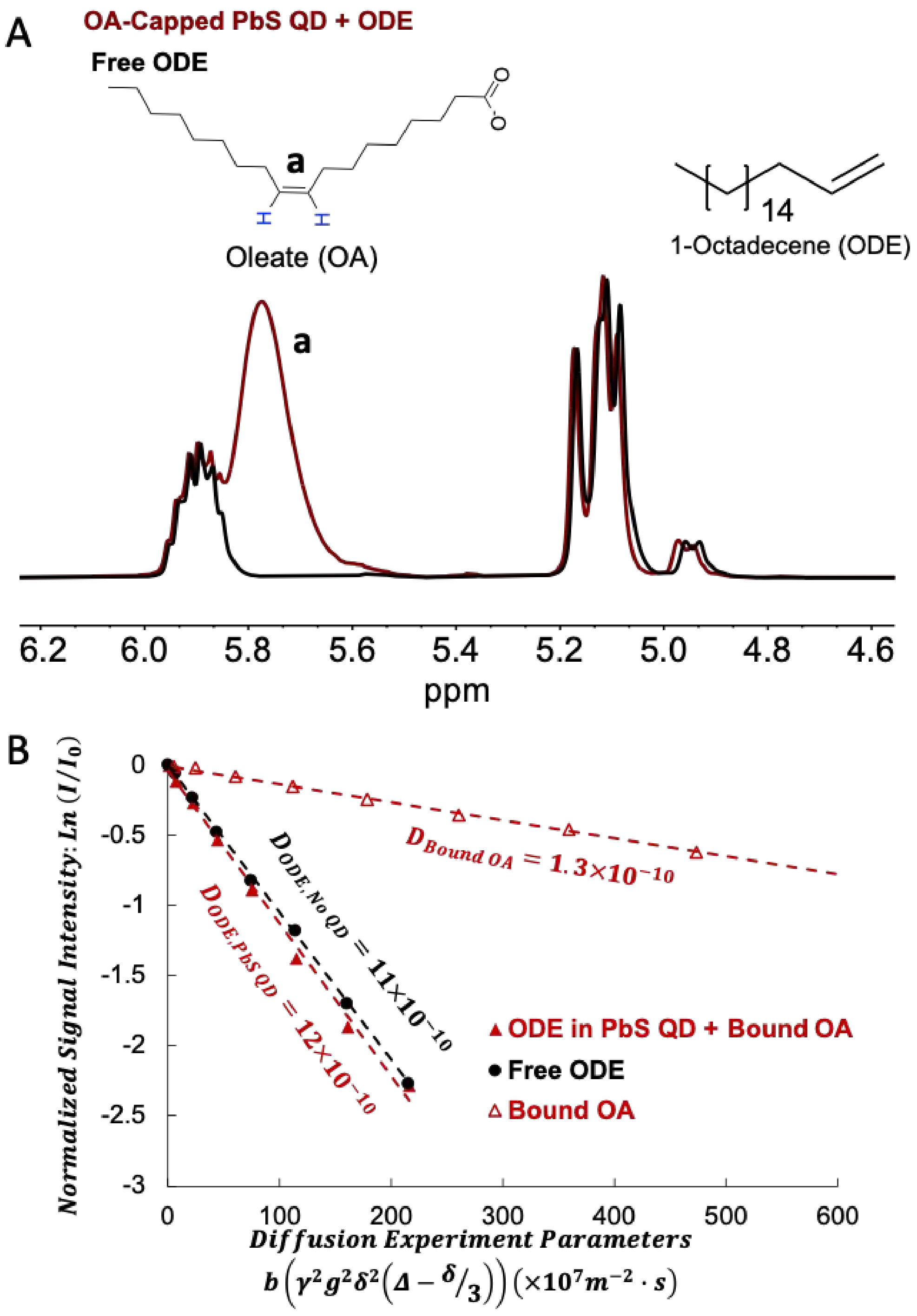

Figure S5: **A**. ^{1}H NMR spectrum of free 1-octadecene (ODE) in toluene-d_8 (black) and ODE in a 150 µM 3.6 nm OA-capped PbS QD solution in toluene-d_8 (red). **B**. Diffusion coefficient ($m^2 \cdot s^{-1}$) of ODE in both solutions (black circle, red triangle) are measured using NMR diffusometry. The diffusion coefficient of the *S_Bound* OA is also measured as shown by red hollow triangle data.

4. ^{1}H NMR Spectra of Oleic Acid Titrated into an OA-capped PbS QD Solution.

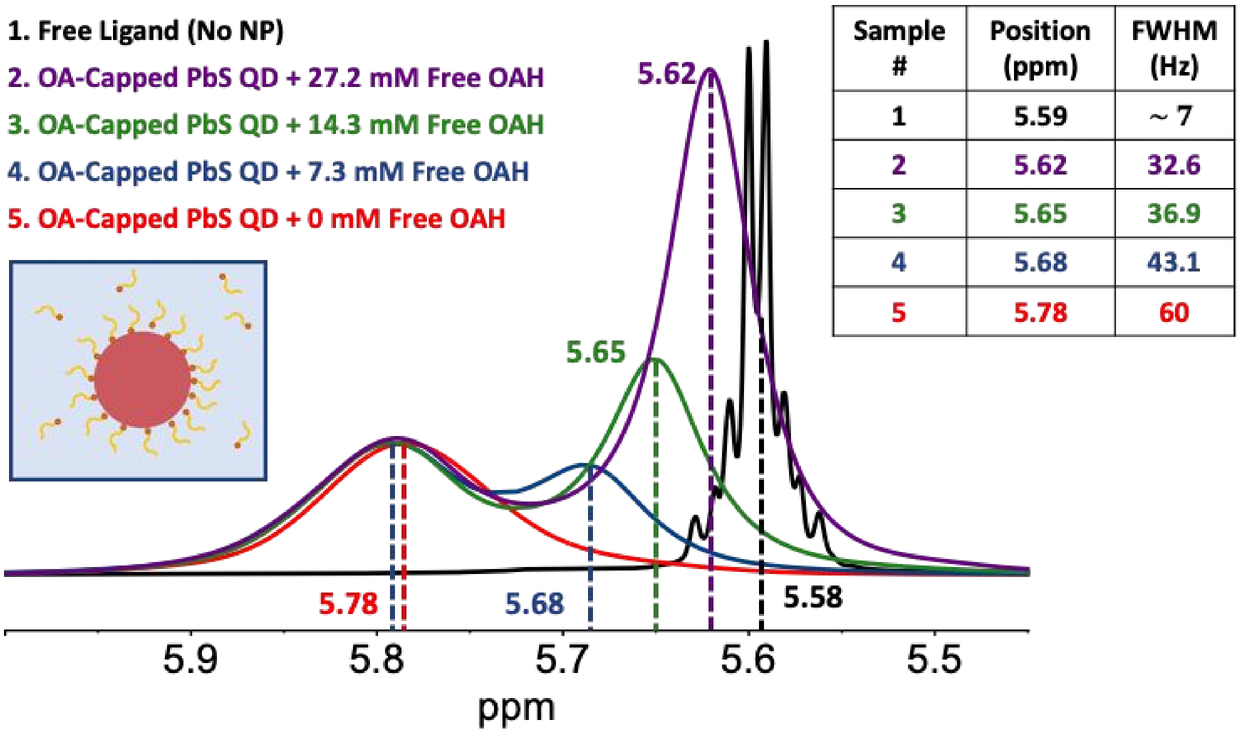

Sample #	Position (ppm)	FWHM (Hz)
1	5.59	~ 7
2	5.62	32.6
3	5.65	36.9
4	5.68	43.1
5	5.78	60

Figure S6: ^{1}H NMR spectra of 150 μM 3.6 nm OA-capped PbS QDs titrated with increasing concentration of oleic acid (OAH) in toluene-d_8 using a 600 MHz instrument. A downfield shift of the exchanging OAH signal (sample #2, #3, #4) and increase in full width at half maximum ($FWHM$, values in the table) with increased OAH titration concentration in the PbS QD solution is observed. The spectra also include a free OAH only sample in toluene-d_8 (# 1, black) and a OA-capped PbS QD sample with no additional OAH titrated in toluene-d_8 (#5, red) along with their peak widths (Hz) and chemical shifts listed in the table.

The above ^{1}H NMR measurements were performed on a 600 MHz Bruker NMR instrument. The S_Bound oleate (OA) ligand signal (5.78 ppm) is normalized across all samples except for the free OAH ligand (#1, black trace). We observe an upfield shift in the exchanging signal from 5.68 ppm to 5.62 ppm as the concentration of titrated OAH increases in the solution. This shift indicates that the chemical shift of the exchanging signal depends on the population fractions of W_Bound and $free$ OAH ligand (**Equation 3** from manuscript). As the concentration

of the free ligand in the fast exchange increases, the exchanging signal's chemical shift moves toward the non-exchanging free ligand's chemical shift (5.58 ppm) as shown in **Figure S6.**

5. PbS QD and Ligand Shell Size Determination Using NMR Diffusometry

We measured the hydrodynamic diameter ($d_{H,\,QD}$) of PbS QDs at room temperature (23 °C) using NMR diffusometry and the Stokes-Einstein Equation

$$D_{QD} = \frac{2kT}{c\pi\eta d_{H,\,QD}} \qquad \text{S2}$$

where k is the Boltzmann constant, T is the temperature, D_{QD} is the diffusion coefficient of the OA-capped PbS QDs, η is the bulk solution viscosity, and $c\pi$ is a prefactor, which is 6π for very large spherical particles diffusing in a surrounding medium of small molecules.[2]

The diffusion coefficient of pure toluene measured at 23 °C is 2.21×10^{-9} $m^2\cdot s^{-1}$ and its measured viscosity is 0.575 mPa·s.[3] We assume the hydrodynamic radius of toluene remains unchanged in the QD solution since the low QD concentration does not significantly affect toluene's molecular structure. However, the introduction of OA-capped QDs may alter the bulk solution viscosity, which we determine by measuring the diffusion coefficient of toluene in solution using the relationship $\eta D = constant$.

Table S1 presents the measured diffusion coefficients of toluene and the corresponding viscosities for solutions with varying OAH titration concentrations. The measured diffusion coefficients fall within the error limits of the diffusometry experiments, indicating that the viscosity remains close across all samples.

Table S1: Diffusion coefficient of toluene in the 150 μM 3.6 nm OA-capped PbS QD solutions titrated with excess OAH. The viscosity of the QD solutions does not change appreciably with increase in excess OAH in the solution.

Sample	Toluene Diffusion Coefficient ($m^2 \cdot s^{-1}$)	Viscosity ($mPa \cdot s^{-1}$)
0 mM OAH (No QD)	$22.1 \pm 1.1 \times 10^{-10}$	0.58 ± 0.03
150 μM QD + 7.3 mM OAH	$21.6 \pm 1.1 \times 10^{-10}$	0.59 ± 0.03
150 μM QD + 14.3 mM OAH	$21.8 \pm 1.1 \times 10^{-10}$	0.58 ± 0.03
150 μM QD + 27.2 mM OAH	$20.5 \pm 1.1 \times 10^{-10}$	0.62 ± 0.03

Based on NMR Diffusometry measurements (**Table S2**), the diffusion coefficients of the PbS QDs remained constant despite increasing the titration concentration of OAH. The observed changes in diffusion coefficients are within the error of the diffusion coefficient measurement. This consistency suggests that the particle diameter (QD core + ligand shell) remains unchanged. This experimental evidence further suggests that only a monolayer of ligands binds to the PbS QD surface (**Figure S7**). Using **Equation S2**, we determined the hydrodynamic diameter of free OAH ($d_{H,\, lig}$), allowing us to determine the QD core diameter (d_{QD}) to be $= 3.6 \pm 0.4\ nm$. Notably, the histogram obtained from TEM imaging resulted in a measured QD diameter of $2.6 \pm 0.3\ nm$. The discrepancy between TEM and NMR measurements falls within their respective error limits of the measurements.

Several factors may contribute to this discrepancy. Firstly, the pre-factor ($c\pi$) for a free ligand molecule is expected to be lower than 6π, as the ligand is slightly larger than the surrounding toluene-d_8 solvent medium. This affects the accuracy of size estimation *via* NMR. Secondly, our assumption that the ligand conformation remains unchanged in both solvent and near the QD surface may not be entirely accurate. If the ligand transitions from a random coil in solution to an

extended conformation when bound to the QD, this could lead to a smaller core particle size determined from NMR measurements. However, the precise nature of these conformational changes remains uncertain and is outside the scope of this work.

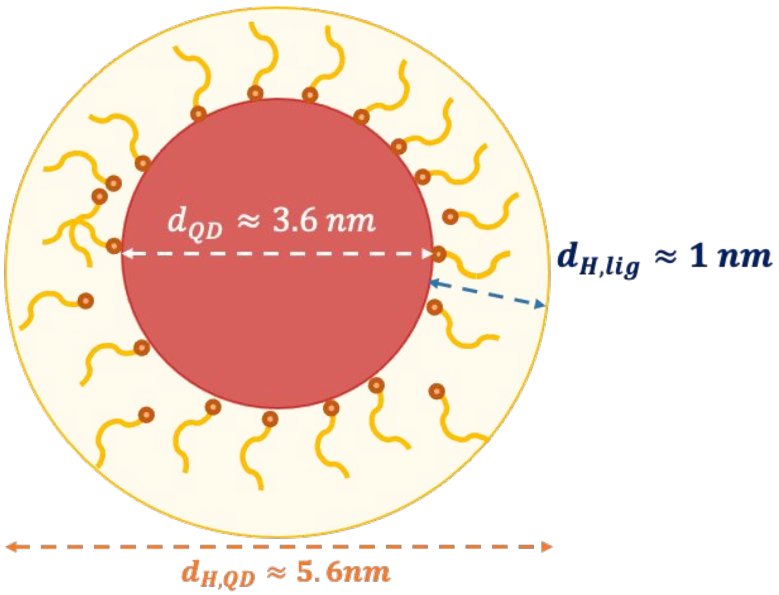

Figure S7: Hydrodynamic diameter of OA-capped PbS QDs and the diameter of the free OAH. These diameters are determined using the Stokes-Einstein equation (**Equation S2**).

Table S2: Diffusion coefficients of 3.6 nm OA-capped PbS QDs (D_{QD}) and free OAH (D_{lig}) in toluene-d$_8$ and their hydrodynamic diameters ($d_{H,QD}$, $d_{H,\,lig}$) measured using **Equation S2**.

	Diffusion Coefficients ($10^{-10}m^2/s$)		Hydrodynamic Diameter (nm)	
Sample	D_{QD}	D_{ligand}	$d_{H,\,QD}$	$d_{H,\,ligand}$
QD + 0 mM OAH	1.3 ± 0.1	7.1 ± 0.2	5.6 ± 0.4	1.0 ± 0.1
QD + 7.3 mM OAH	1.2 ± 0.1	7.1 ± 0.2	6.2 ± 0.4	1.0 ± 0.1
QD + 14.3 mM OAH	1.3 ± 0.1	7.1 ± 0.2	5.6 ± 0.4	1.0 ± 0.1
QD + 27.2 mM OAH	1.3 ± 0.1	7.1 ± 0.2	5.6 ± 0.4	1.0 ± 0.1

The diffusion coefficients and hydrodynamic diameter of the OA-capped PbS QDs remained same in all samples indicating a monolayer of OA ligands on the QD surface.

6. Total Number of Pb Atoms on the Surface of 3.6 nm OA-capped PbS QDs

A 3.6 nm PbS QD was modeled using the Visualization for Electronic and Structural Analysis (VESTA) software. The PbS unit cell (mp-21276) was retrieved from The Materials Project and set to a cubic system with space group (F m –3 m). The boundary of the system was adjusted to 6 in the xyz maximum and minimum values. The crystal shape setting was used to model the faceting of the QD surface. For a modeled PbS QD, the diameter can be calculated by the number of repeating lattice units (Pb–S–Pb) multiplied by the lattice parameter $a = 0.593\ nm$.[4] Thus, a total of six repeating lattice units are needed to maintain a 3.6 nm diameter QD.

Within this limitation, three QD models were made with varying areas of (111) and (100) facets that fulfill the requirement of (6) x (0.593 nm) = 3.6 nm (**Figure S8**). The relative facet areas were adjusted in the models by changing the distance of from origin of the (111) facet (4 x d from origin, 5 x d from origin and 6 x d from origin for models **1**, **2** and **3** respectively) relative to the distance from origin of the (100) facet (3 x d from origin for all model). Any changes to the distance from origin of the (100) facet relative to the (111) facet resulted in a change in lattice repeats and thus modeled QD diameter.

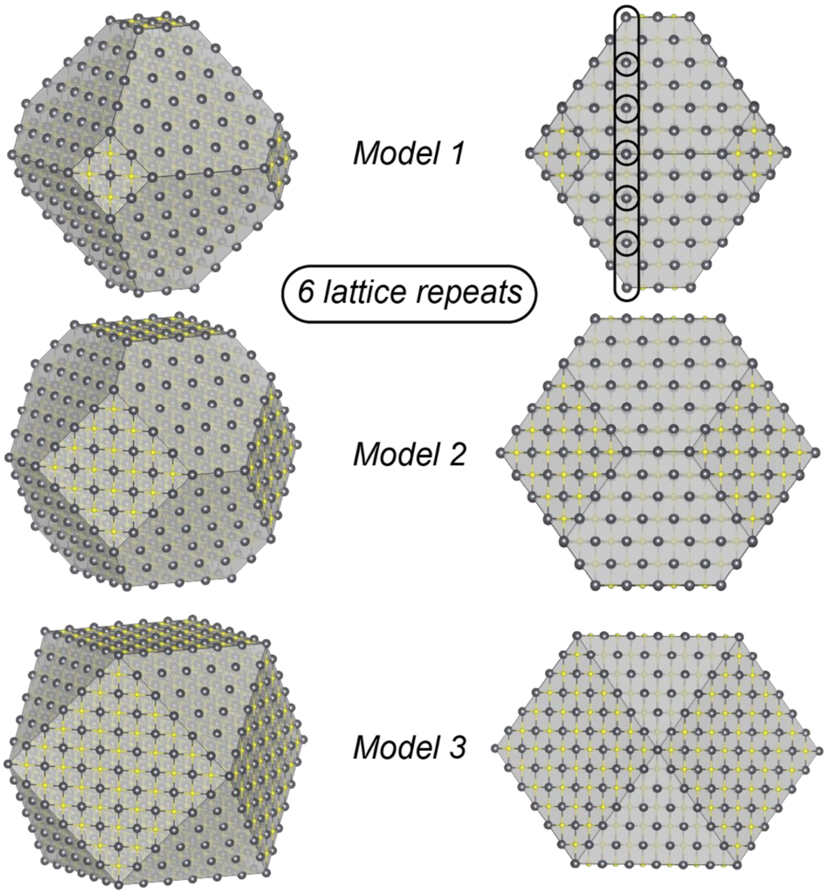

Figure S8: VESTA models of a 3.6 nm PbS QD with three different relative (111) and (100) facet areas. The rounded rectangles emphasize the areas that are counted when considering the Pb–S–Pb lattice repeat unit, which total 6 for the models presented here.

With the three models presented, we calculated the total number of Pb sites in each one of the facets without including the (111)/(100) edge sites (**Table S3**). Correlating the total number of Pb sites in the facets with the total number of S_Bound and W_Bound ligands quantified to saturate the QD surface through ^{1}H NMR spectroscopy (**Table S5**), we identified model **2** to be the most representative model for our system. At room temperature, the surface coverage of S_Bound and W_Bound ligands reached a maximum of 191 ligands at 27.2 mM OAH, which is consistent with the total number of 198 Pb sites on the (111) and (100) facets calculated with model **2** without including the (111)/(100) edge sites. When accounting for the 108 Pb atoms on the (111)/(100)

edges in model **2**, a total of 306 surface Pb atoms are quantified, which aligns closely with the 190 S_Bound and 77 W_Bound ligand saturation at $-10\ ℃$ (total ligand coverage = 264) and considers residual Pb on the surface where OA or OAH do not bind. Here we note that edge sites may count as either X-type (S_Bound) or L-type (W_Bound) binding sites, so from our NMR measurements it appears more likely that edge sites will bind as L-type. Furthermore, the complexity of charge balance and potential excess Pb atoms on (111) surfaces could account for the higher approximate saturation value of 190 X-type ligands observed by NMR at -10 °C and 27.2 mM OAH addition.

Table S3: Estimated number of Pb atoms on the surface of a 3.6 nm PbS QD modeled using VESTA with different relative (111) and (100) facet areas. The total number of Pb sites do not include the number Pb atoms on the (111)/(100) edges, which equals 108 for Model 2.

Model	# of (111) facets	# of Pb atoms on a (111) facet	Total # of Pb atoms on all (111) facets	# of (100) facets	# of Pb atoms on a (100) facet	Total # of Pb atoms on all (100) facets	Total # of Pb sites on all facets
**1**	8	18	144	6	1	6	150
**2**	8	18	144	6	9	54	198
**3**	8	10	80	6	25	150	230

To further support the assignment of 306 surface Pb atoms at the surface modeled with VESTA, the total number of Pb atoms at the surface was estimated mathematically using the approximation by Valdez et al.[5] Bulk PbS has a rock salt (face-centered-cubic, FCC) structure with each unit-cell face containing two Pb^{2+} and one S^{2-} atom (**Figure S9**). To determine the number of Pb surface atoms, we assume that the QD has an FCC packing structure. The atomic radii of Pb^{2+} and S^{2-} ($r_{Pb^{2+}}$ and $r_{S^{2-}}$) are 0.12 and 0.18 nm respectively[1], giving us the lattice parameter of the

unit cell $a_{unit} = 0.6 \pm 0.04\ nm$. The number of atoms on the QD surface can be determined from **Equations S3-S5**

$$Area\ of\ PbS\ (100)\ unit\ cell\ face = a_{unit}^2 = 0.36 \pm 0.025\ nm^2 \qquad S3$$

$$Surface\ area\ of\ 3.6\ nm\ QD = \pi d_{QD}^2 = 40.7 \pm 6\ nm^2 \qquad S4$$

$$Total\ Number\ of\ Surface\ Atoms\ on\ a\ QD\ particle = \frac{4 \times \pi d_{QD}^2}{a_{unit}^2} \approx 450 \pm 80 \qquad S5$$

Thus, a 3.6 nm QD has a total of 450 ± 80 atoms (Pb + S) on the surface. However, PbS QDs exhibit a non-stoichiometric behavior at the surface which was studied by Rutherford backscattering spectroscopy (RBS)[1] where the ratio of Pb:S atoms depend on the size of the QD. For a QD with a size of 3.6 nm the Pb:S ratio measured was 1.37:1. With this factor in consideration, the number of Pb atoms on the surface is approximately 260 ± 50 atoms.

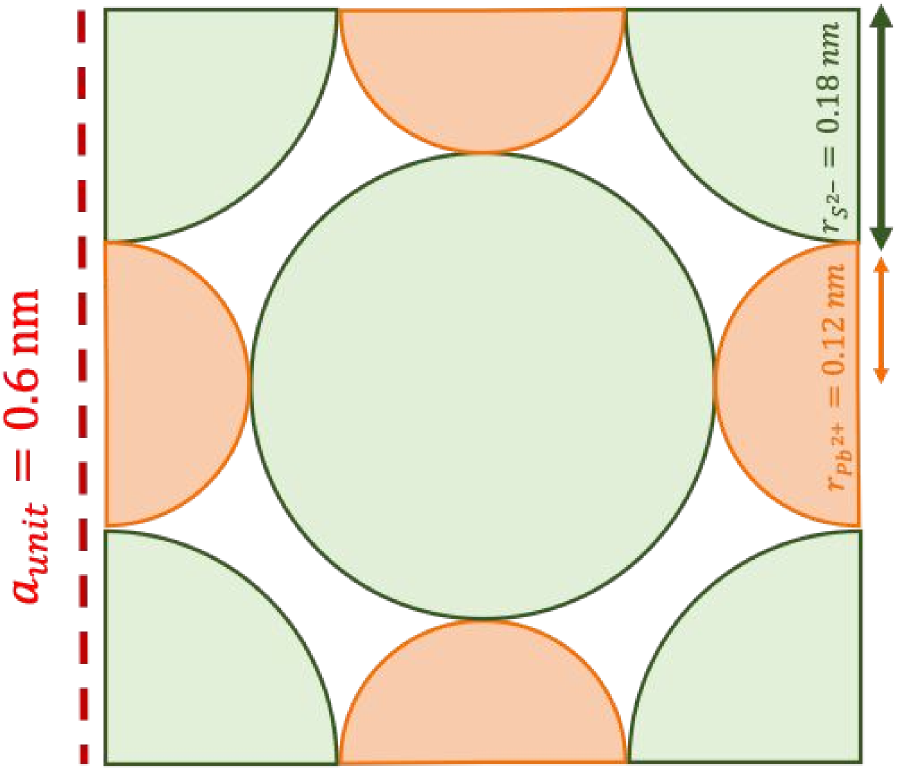

Figure S9: Face of a rock salt PbS unit cell having face-centered-cubic packing. The atomic radii of Pb^{2+} and S^{2-} are 0.12 and 0.18 nm respectively and the lattice parameter $a_{unit} = 0.6 \pm 0.04\ nm$.

7. Determination of Average Number of Ligands in Different States Per PbS QD

We quantified the number of ligands per QD by adding ferrocene as an internal standard to the NMR solution. Approximately $1\,m$M of ferrocene was added to a toluene solution containing $0.15\,mM$ of QDs with unknown concentration of S_Bound oleate (OA) ligands stabilizing the QDs (see the Methods section in the manuscript for details). Specifically, for each sample, 590 µL of a QD stock solution was transferred to an NMR tube and 10 µL of the ferrocene internal standard added to achieve the desired 0.15 mM QD concentration in 600 µL. To probe the ligand exchange process, we prepared additional samples by titrating the 0.15 mM QD solution with 7.3, 14.3 and 27.2 mM of oleic acid (OAH) (corresponding to $50\times$, $100\times$, $200\times$ the QD concentration, respectively). Each sample was prepared under inert N_2 conditions and flame sealed into glass to ensure long-term stability of the synthesized QDs.

For accurate quantification of molecular concentration, it is crucial to use an NMR pulse sequence with a long relaxation delay time (t_{rd}) to allow complete relaxation of spin magnetic moments back to the longitudinal z-axis. The recommended repetition time (T_R) for each scan should be greater than 5 times the largest spin-lattice relaxation time ($T_{1,\,largest}$) as shown in **Equation S6**.

$$T_R = t_{aq} + t_{rd} > 5\ \times T_{1,\,largest} \qquad \text{S6}$$

For optimal signal-to-nose ratio, we set an acquisition time $t_{aq} = 2\,s$, while adjusting t_{rd} according to the measured $T_{1,\,largest}$ of the solution. In inert conditions (N_2 atmosphere), relaxation times tend to be longer due to the absence of O_2 mediated dipolar interactions, which otherwise provide an additional relaxation pathway. The measured spin-lattice relaxation time constant T_1 for the ferrocene protons was 30 s and 1 s for the oleic acid alkenyl protons.

To ensure $> 99.9\%$ relaxation, we applied a 15° pulse angle time and set $t_{rd} = 100\,s$ between scans. After acquisition, signals were phase-corrected and integrated to quantify moles of OA+OAH ligand ($Moles_{OA+OAH}$) relative to the ferrocene standard using **Equation S7**:

$$Moles_{OA+OAH} = 5 \times Moles_{ferrocene} \times \left(\frac{IA_{OA+OAH}}{IA_{ferrocene}}\right) \qquad \text{S7}$$

Where IA_{OA+OAH} and $IA_{ferrocene}$ are the integral areas of the OA+OAH peak ($6.0 - 5.4\,ppm$) and the ferrocene peak ($4.1\,ppm$) respectively (see **Figure S10**). Since ferrocene (4.1 ppm) contributes 10 aromatic protons and OA ligands ($5.8\,ppm$) contribute 2 alkenyl protons, we apply a multiplication factor of 5 to obtain the ligand (OA+OAH) concentration (**Figure S10**).

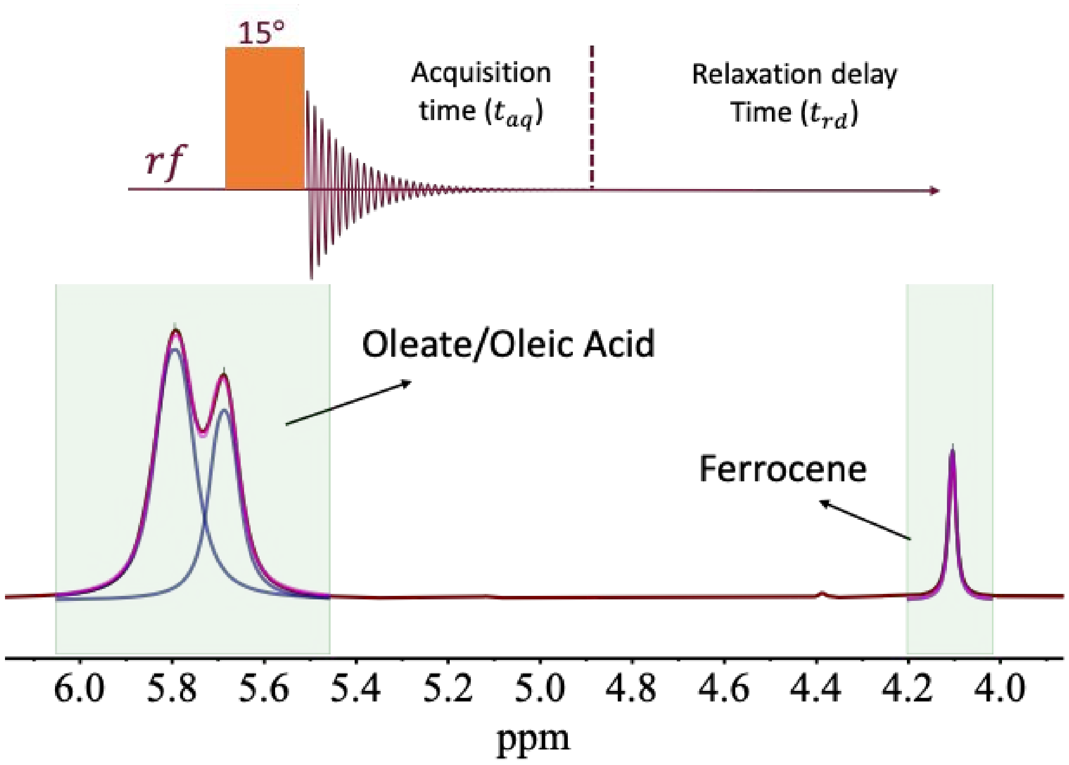

Figure S10: Quantifying OA/OAH concentration in a 150 µM 3.6 nm OA-capped PbS QD solution using a ferrocene internal standard and quantitative-NMR technique.

Table S4 lists the number of ligands (OA+OAH) per QD for four samples. The first sample contains only S-Bound oleate (OA) on the QD surface. The remaining three samples were titrated

with increasing concentration of OAH. The concentration of ferrocene and QDs were known when the samples were prepared (more details in the Methods section of the manuscript), and the concentration of OA+OAH is determined using **Equation S7**.

Table S4: Quantification of ligands (OA+OAH) per QD using 1D ^{1}H NMR spectroscopy. By integrating the ferrocene and the ligand signals and applying **Equation S7**, we determine the total ligand count per QD.

Sample	OA+OAH Integral	Ferrocene Integral	Ferrocene (μMoles)	QD (μ Moles)	OA+OAH (μMoles)	OA+OAH per QD
OA-capped PbS QD Only	1	0.2106	0.60	0.09	14.2	158
QD + 7.3mM OAH	1	0.1446	0.58	0.09	20.0	222
QD + 14.3 mM OAH	1	0.1142	0.57	0.09	25.1	279
QD + 27.2 mM OAH	1	0.0829	0.57	0.09	34.6	385

We also studied the changes in population of OAH and OA in each state with increased titration concentration of OAH with respect to temperature and were able to determine their populations (absolute number of ligands) in each state per QD as shown in **Table S5**.

Table S5: Quantified number of ligands in each state per 3.6 nm OA-capped PbS QD for samples containing varying concentration of OAH titrated in the solution. Quantified at temperatures from 23 °C to -10 °C

Number of Ligands per PbS Quantum Dot Particle			
OA-capped PbS QD Only	QD + 7.3 mM OAH (Total # = 222)	QD + 14.3 mM OAH (Total # = 279)	QD + 27.2 mM OAH (Total # = 385)

Temperature (ºC)	Bound	S_Bound	W_Bound	Free	S_Bound	W_Bound	Free	S_Bound	W_Bound	Free
23	158 ± 20	141	58	23	127	62	90	120	71	194
10	158	159	44	19	138	66	75	156	77	152
0	158	175	31	17	160	75	44	150	77	158
–10	158	173	41	8	166	74	39	190	74	121

S_Bound = $[Occupied\ S\_Bound\ Sites]$, W_Bound = $[Occupied\ W\_Bound\ Sites]$, Free = $[Free\ Ligand]$

8. Chemical Shift of W_Bound OAH Ligand from Ligand Population Fractions

Table S6: Chemical shift of the W_Bound ligand determined using **Equation 2**. The chemical shift of the W_Bound ligand indicates that the W_Bound and S_Bound ligands have a similar chemical environment. Hence it is accurate to assume that the diffusion coefficients of instantaneous W_Bound and S_Bound ligands are same.

	Ligand Population Fraction Contributed to Fast Exchange		Chemical Shifts of Ligands			
Sample	$Free$ (%)	W_Bound (%)	S_Bound	$Exchanging$	$Free$	W_Bound
OA-capped PbS QD Only	N/A	N/A	**5.78**	N/A	5.58	N/A
QD + 7.3 mM OA	28.5	71.5	**5.79**	5.69	5.58	**5.74**
QD + 14.3 mM OA	59.0	41.0	**5.79**	5.65	5.58	**5.76**
QD + 27.2 mM OA	73.2	26.7	**5.79**	5.62	5.58	**5.76**

9. Thermodynamic Parameters Determined From the Exchange Equilibrium Constant

To further investigate the energetics of weak and strong ligand binding on the QD surface, we determined their equilibrium constants (K_{S_Bound}, K_{w_Bound}), using **Equation 5** and **6**, at four different temperatures (23 °C to –10 °C). Initially, K_{S_Bound}, K_{w_Bound} were determined based on the surface-bound ligand populations (~ 190 OA + 77 OAH) (see **Table S5**), assuming complete saturation of $[Available\ W(100)\ Sites]$ and $[Available\ S(111)\ Sites]$ per QD. But this led to equilibrium constants approaching infinity, particularly for the sample with 27.2 mM of OAH at

-10 ℃, suggesting that not all Pb sites are saturated by OA/OAH ligands. This observation aligns with total number of Pb sites estimated from the VESTA model. To correct this discrepancy, additional ~ 20 Pb sites were equally distributed between facets, yielding 210 $[Available\ S(111)\ Sites]$ and 100 $[Available\ W(100)\ Sites]$ per QD, adding up to the total of 310 surface Pb atoms quantified for a QD of size 3.6 nm from the VESTA and Valdez's model.[5] The final K_{S_Bound} and K_{w_Bound} values, based on these adjusted assumptions, are listed in **Table 2** of the manuscript.

The Van't Hoff plots derived from the equilibrium constants and temperature variations provide enthalpic (ΔH) and entropic ($T\Delta S$) energy changes for both ligand binding motifs (**Figure S11**). Since the change in W_Bound OAH ligand populations are within the error of the measurements, the W_Bound ligand population changes are inconsistent, leading to greater error in the K_{w_Bound} measurement and a lack of clear trends in ΔH_{W_Bound} ($-21.4 \pm 13.6\ KJ/mol$) with increasing OAH concentration (**Figure S11B**). In contrast, the errors for K_{S_Bound} are smaller, as the temperature dependent S_Bound ligand population changes are greater than the measurement error. Consequently, we observe consistent enthalpic changes for S_Bound ligands ($\Delta H_{S\_Bound} = -38.7 \pm 3.0\ KJ/mol$) as shown in **Figure S11A**.

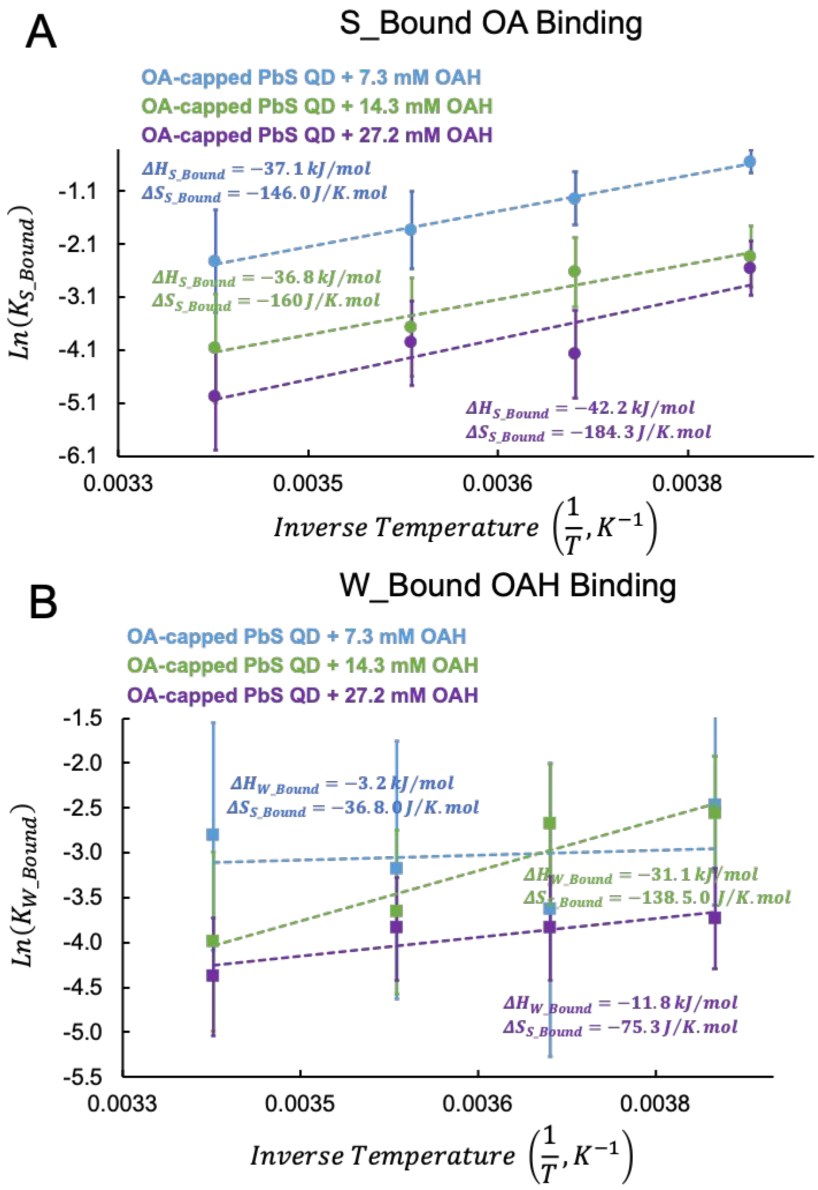

Figure S11: Van't Hoff plots of (**A**) OA ligand binding on (111) and (**B**) OAH ligand coordination on (100) facets. The entropy (ΔS) and enthalpy (ΔH) changes for these reactions are shown in the inset.

The enthalpy (ΔH), entropy ($T\Delta S$), and Gibbs free energy (ΔG) changes for both ligand binding motifs (S_Bound and W_Bound) at room temperature (25℃) as a function of OAH titration concentration are shown in **Figure S12**. Both weak and strong ligand binding processes are exothermic, as indicated by the negative ΔH values. Notably, the enthalpy change for S_Bound

OA binding is more negative than for W_Bound OAH binding ($\Delta H_{S\_Bound} < \Delta H_{W\_Bound}$), suggesting that the OA ligand binding on the (111) facet is stronger than OAH ligand binding on the (100) facet. These results are in accordance with the to the *ab initio* calculations by Wang *et al*. and are consistent with the NMR spectroscopy measurements where the bound signal did not change after titrating OAH, indicating stronger binding of S_Bound OA ligands compared to W_Bound OAH.[6]

The entropic penalty for ligand binding should be greater for the S_Bound OA ligands compared to W_Bound OAH ligands ($\Delta S_{W\_Bound} < \Delta S_{S\_Bound}$), due to the higher surface density of Pb^{2+} ions on the (111) facet compared to the (100) facet. This should lead to denser ligand packing on the (111) facet, resulting in a greater entropic cost for S_Bound ligand binding. These determined thermodynamic values further suggests that the OA/OAH ligand binding are enthalpy-driven, making their coverage on the QD surface more favorable at lower temperatures.

The small positive Gibbs free energy for S_Bound (ΔG_{S_Bound}) OA ligands indicates that OA ligands do not fully saturate the $Available\ S(100)\ Sites$, suggesting that OA ligand binding on the QD surface is not spontaneous. Additionally, as excess OAH is titrated into the solution, ΔG_{S_Bound} increases, further reducing spontaneity. This insight highlights oleic acid's suitability for ligand exchange reactions, as its binding process is reversible. In contrast, the Gibbs free energy for W_Bound OAH ligands is close to zero, indicating a fast equilibrium between W_Bound and $free$ OAH ligands – an observation consistent with NMR spectroscopy and diffusometry measurements.

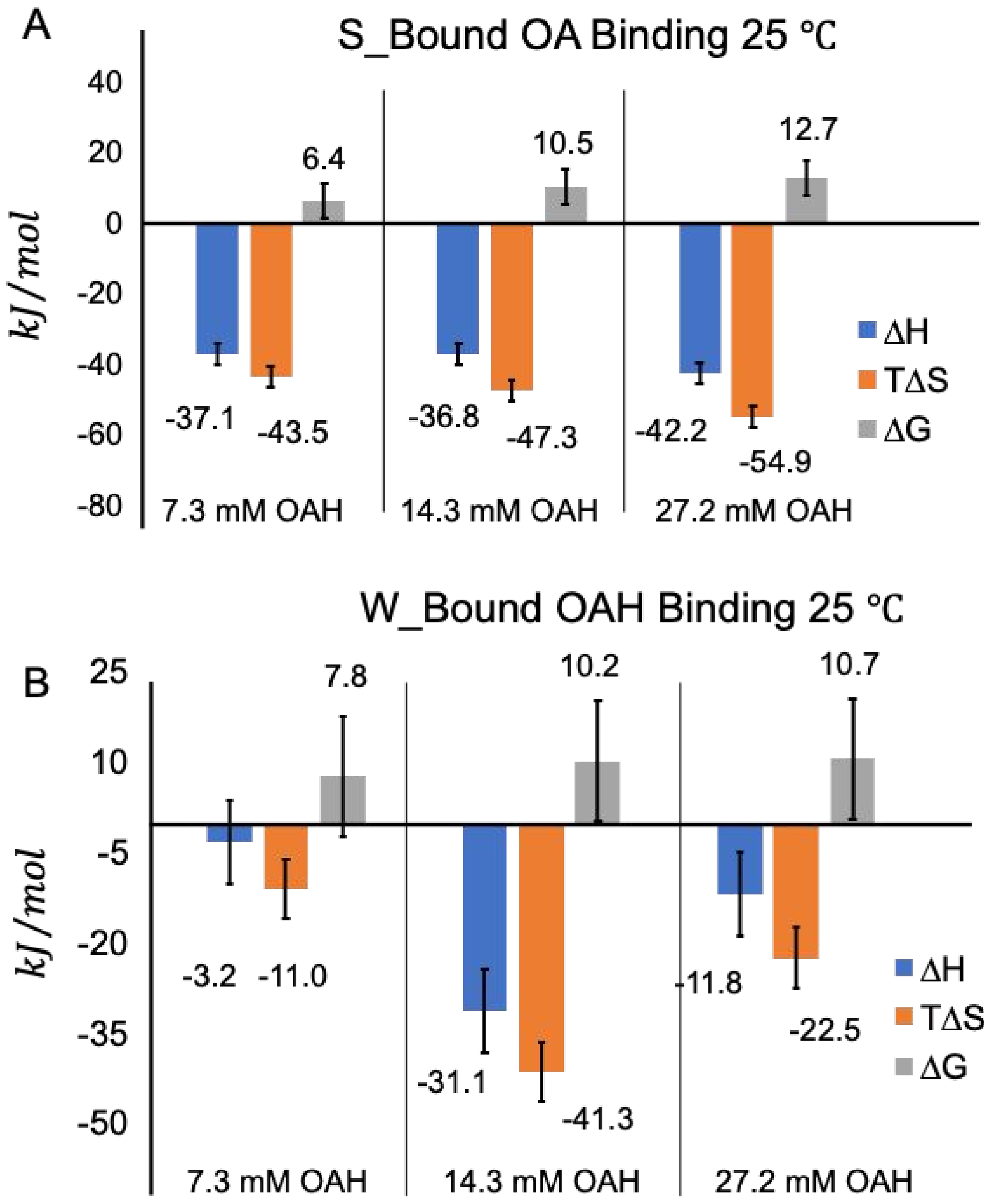

Figure S12: **A, B.** The enthalpy (ΔH), entropy (ΔS), and Gibbs (ΔG) free energy for weak and strong ligand binding ($S - Bound$ and $W - Bound$) for all three titration concentrations of free OAH at 25°C. These processes are enthalpy-favored reactions and the positive ΔG indicates that the ligand binding on the QD surface reversible at room temperature making oleic acid ideal ligand for ligand exchange reactions.

10. Understanding Exchange Kinetics From Dynamic NMR Spectroscopy

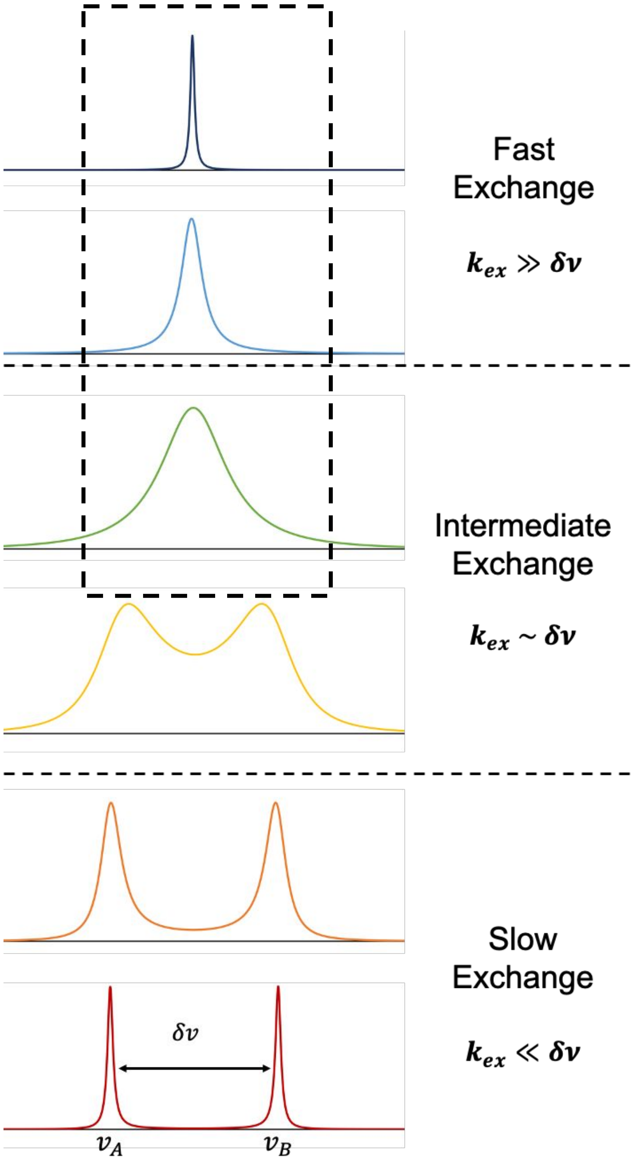

Figure S13: Understanding two-state exchange kinetics of a molecule using dynamic NMR spectroscopy. The exchanging process can be classified in three regimes which is dependent on the exchange rate (k_{ex}) and chemical shift difference (δv) between the two states that are exchanging. For our system, the observed exchange regime is highlighted in the box.

In a slow exchange regime, we observe two distinct narrow NMR signal peaks of both molecular states (v_A, v_B). The width of the peak broadens when the exchange rate increases as shown in **Figure S13**. In an intermediate exchange regime, the signal peaks begin to coalesce into a single peak. In a fast exchange regime, the coalesced single exchanging peak further sharpens

indicating the nuclei of the probed moleucle is in very fast exchange between both states. The exchange regimes depends on the chemical shift difference $\delta v = v_A - v_B$ of the moleucle in two states that are not exchanging and the exchange rate k_{ex} of the molecule between two states. For our system, the exchange regime is transitioning from intermediate to fast exchange as the temperature increases from -10 to 23°C as highlighted by the box. **Figure S14** below further indicates the change in integral and $FWHM$ of the exchanging signal with respect to temperature for a sample containing 3.6 nm OA-capped PbS QDs with 14.3 mM of excess oleic acid (OAH) titrated into the solution. These changes further indicates the change in dynamics and kinetics of ligand binding to the PbS QD surface.

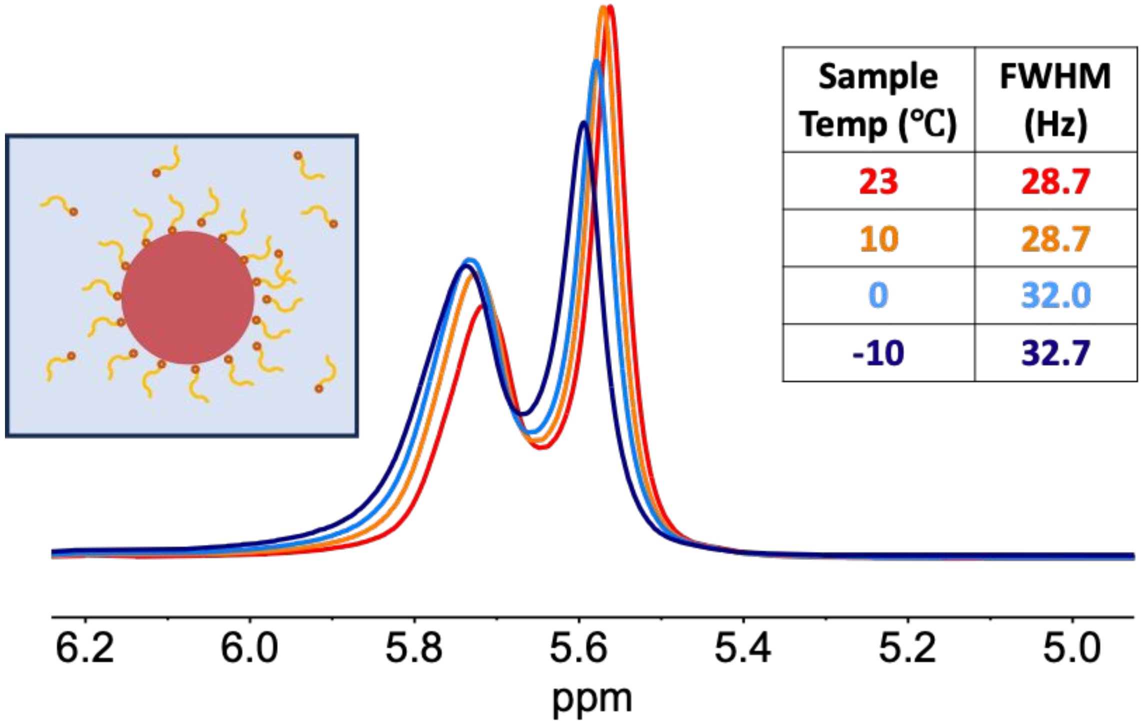

Figure S14: Changes in $FWHM$, integral, and chemical shift of the exchanging signal (v_{ex}) for the alkenyl proton as the temperature decreases from 23°C to -10°C in a 150 µM 3.6 nm OA-capped PbS QD solution titrated with 14.3 mM OAH.

11. Spin-spin Relaxation Time Constant T_2 Measurements of Free Oleic Acid and S_Bound Oleate Ligand in Toluene-d$_8$ Solution.

The spin-spin relaxation time T_2 for $free$ oleic acid and for S_Bound oleate (OA) were measured using a modified CPMG (Carl-Purcell-Meiboom-Gill) pulse sequence to compensate for J modulation that significantly affects T_2 values measured using a standard CPMG experiment. The pulse sequence for the experiment is given in **Figure S15** and the pulse program is listed below. This program was initially developed by Dr. Gareth A. Morris' group[7], and we adopted it by adding a 90°$_y$ pulse and same phase cycling to the existing CPMG pulse sequence to reduce the effect of J-modulation. **Table S7** lists the spin-lattice relaxation time of S_Bound and $free$ ligands with respect to temperature.

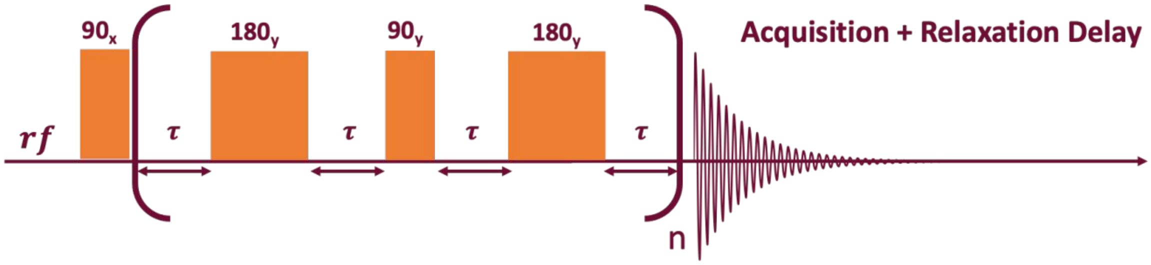

Figure S15: Modified CPMG pulse sequence to reduce the effect of J-modulation. Achieved by inserting a 90°$_y$ pulse between spin echoes.

Modified CPMG Pulse Sequence to Compensate for J-modulation

```
;avance-version (12/01/11)
;T2 measurement using Carr-Purcell-Meiboom-Gill sequence compensated for coupling
;$CLASS=HighRes
;$DIM=2D

#include <Avance.incl>

"p2=p1*2"
"d11=30m"

1 ze
2 d1
```

```
  p1 ph1
3 d20
 p2 ph2
 d20
  p1 ph3
d20
  p2 ph2
d20
 lo to 3 times c
 go=2 ph31
  d11 wr #0 if #0 ivc
  lo to 1 times td1
exit

ph1=0 2 ;90d excitation x -x
ph2=1 ;180 refocus y
ph3=1 1 3 3 ;90d refocus y -y
ph31=0 2 0 2

;pl1 : f1 channel - power level for pulse (default)
;p1 : f1 channel -  90 degree high power pulse
;p2 : f1 channel - 180 degree high power pulse
;d1 : relaxation delay; 1-5 * T1
;d11: delay for disk I/O                              [30 msec]
;d20: fixed echo time to allow elimination of diffusion
;     and J-mod. effects
;vc : variable loop counter, taken from vc-list
;ns: 4 * n
;ds: 16
;td1: number of experiments = number of values in vc-list
;define VCLIST
;d20: d20 should be << 1/J ,but > (50 * P2)
```

Table S7: Temperature dependent spin-spin relaxation time of free ($T_{2,free}$) and S_bound (T_{2,S_Bound}) ligands using modified CPMG experiment

Temperature (°C)	$T_{2,free}$ (s)	T_{2,S_Bound} (s)
-10	0.51	0.030
0	0.62	0.036
10	0.70	0.044
23	0.78	0.053

12. Python Model Fitting of the S_Bound and Fast Exchanging ^{1}H NMR Signal

Due to the overlap of the exchanging and bound peaks (**Figure 2,** also **Figure S6 in SI Section 4**), we deconvoluted the signals using MNova. We fit the separated exchanging signal data using complete lineshape function (**Equation 10**) and the bound signal integrals and line positions (chemical shifts) using the following Lorentzian lineshape function

$$S_{S-Bound}(\nu) = \frac{C_1}{2\pi} \times \left(\frac{\frac{FWHM_{S\_Bound}}{2}}{(\nu - \nu_{S-Bound})^2 + \left(\frac{FWHM_{S\_Bound}}{2}\right)^2} \right) \qquad \text{(S8)}$$

where, ν_{S_Bound} is the resonance frequency (in Hz) of the S_Bound signal, $FWHM_{S\_Bound}$ is the linewidth (in Hz) of the bound signal, and C_1 is a scaling factor. We determined ν_{S_Bound} and $FWHM_{S\_Bound}$ from the deconvoluted S_Bound signal.

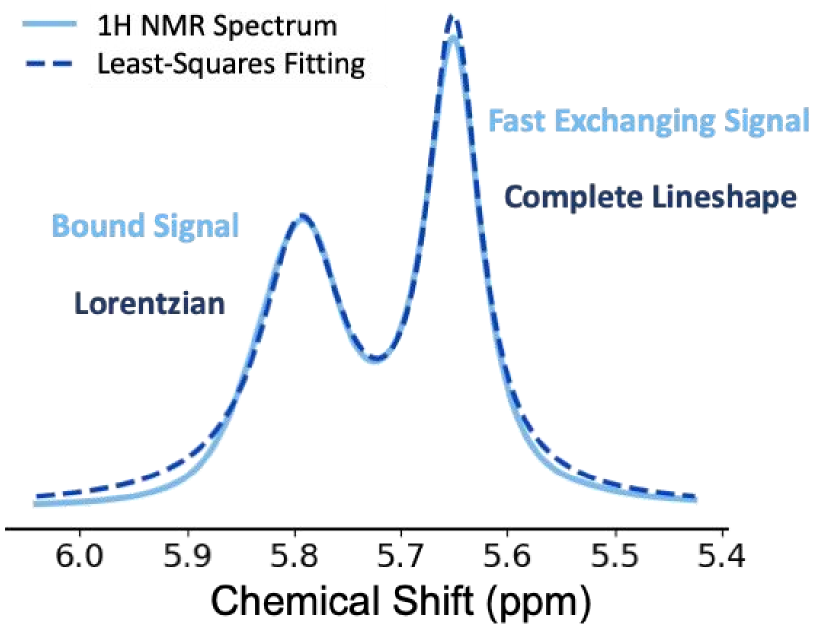

Figure S16: Least-squares fitting (dashed line) of an acquired alkenyl proton spectrum (solid line) for a sample containing 150 μM 3.6 nm OA-capped PbS QDs titrated with 14.3 mM OAH, measured at 23°C. The fitted curve, a linear combination of optimized lineshape and Lorentzian functions, closely matches the acquired data, indicating the accuracy of the determined exchange

lifetime, τ_{ex}. Deviation near the baseline is expected as the NMR spectrum of any sample shows deviation from Lorentzian function.

13. Activation Energy of Slow Ligand Exchange Process

As previously discussed in **Section S1**, NMR can investigate dynamics across multiple time and length scales. To qualitatively estimate the exchange time for strong ligand binding and unbinding to the QD surface (S_Bound), we employed NMR diffusometry measurements. In this measurement, the observed timescale is governed by the diffusion time (Δ). At room temperature (25°C), the alkenyl protons exhibit two distinct signal peaks (near 5.6 and 5.8 ppm) on the spectrum when measured at $\Delta = 25\,ms$. Notably, no changes are observed in the spectrum (FWHM and chemical shift) or the diffusion coefficient of the ligands, even when Δ is increased to $200\,ms$. This observation indicates that the exchange time for S_Bound OA with free OAH is very slow and $> 200\,ms$. Given that this exchange rate is temperature-dependent, we can quantify this dependency using an Arrhenius equation (**Equation S9**)

$$k_{ex,slow} = k_{ex,\infty} e^{-\frac{E_{a,S\_Bind}}{RT}} \qquad \text{S9}$$

Where $k_{ex,\infty}$ is the exchange rate at infinite temperature and E_{a,S_Bind} is the activation energy for the slow exchange between S_Bound OA and free OAH ligands in the solution.

Ligands that undergo both slow and fast exchange processes (S_Bound and W_Bound) transition between the free solvent environment and the restrictive QD surface environment. Despite different ligand interactions on the QD surface, the slow and fast exchange processes share similarities in ligand environments, i.e., in both processes the alkenyl proton nuclei experience similar chemical environment when switched between free and bound states. Given the analogous nature of these processes, we might make a highly simplified assumption that the $k_{ex,\infty}$ for both ligand binding processes are equal. To understand the energetics of the slow exchange process, we

further assumed a lower bound for the S_Bound OA ligand exchange time to be equal to $200\ ms$. From these parametric value assumptions, we can estimate the activation energy for the slow exchange process to be $E_{a,S\_Bind} \approx 100\ KJ/mol$. This value is significantly higher than the activation energy for the fast exchange process ($E_{a,S\_Bind} \approx 5 \times E_{a,W\_Bind}$).

Figure S17 gives a comprehensive overview of the energetics of the oleic acid ligand exchange from the free solvent state to the strong or weakly bound QD surface states, providing information on the binding mechanism and stability of ligands on the QD surface. This can indeed open up new avenues for molecular dynamic (MD) simulations, which can further elucidate the entire exchange process. By leveraging the energetics data through simulations, a more detailed and dynamic understanding of the ligand exchange process will be achieved, leading to improved control over QD surface chemistry, better prediction of QD properties and ultimately, the development of more efficient and stable QD-based devices and applications.

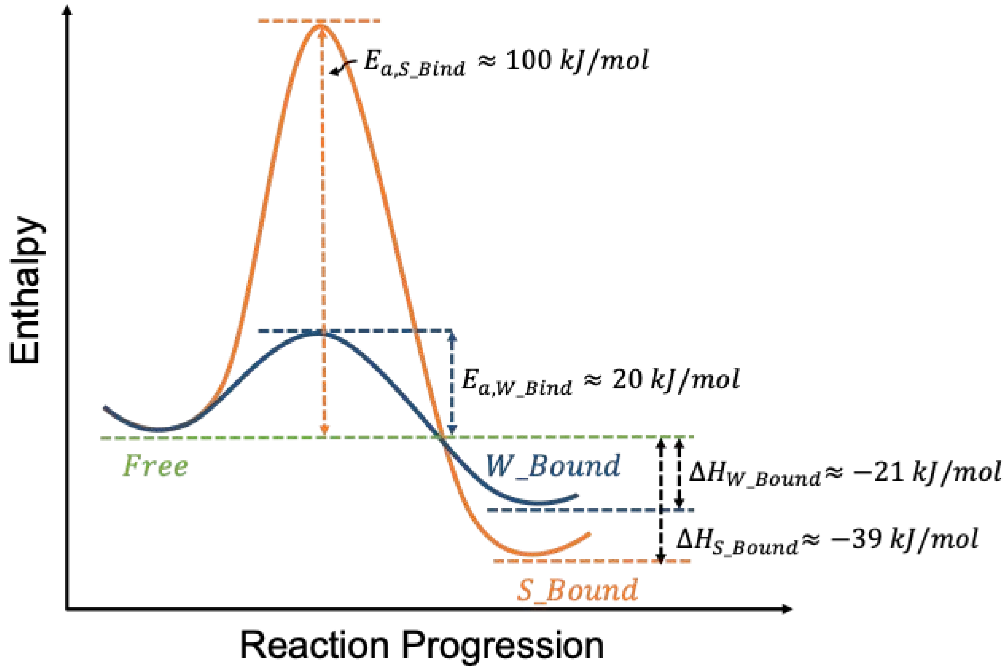

Figure S17: Enthalpy diagrams for strong (S_Bound) and weak (W_Bound) ligand binding on the QD surface. The ΔH for both these processes is differed by a factor 2 (see **Figure S11**) but

their activation energies (E_a) differ by a factor 5. These diagrams clearly indicate the stronger interactions of S_Bound OA ligands and closer packing on (111) facet of the QD surface.

13. ^{1}H NMR Spectrum Comparison of 1D Pulse-Acquire-and PGSTE 1st Slice of OA-capped PbS QDs Titrated with 7.3 mM of Free OAH

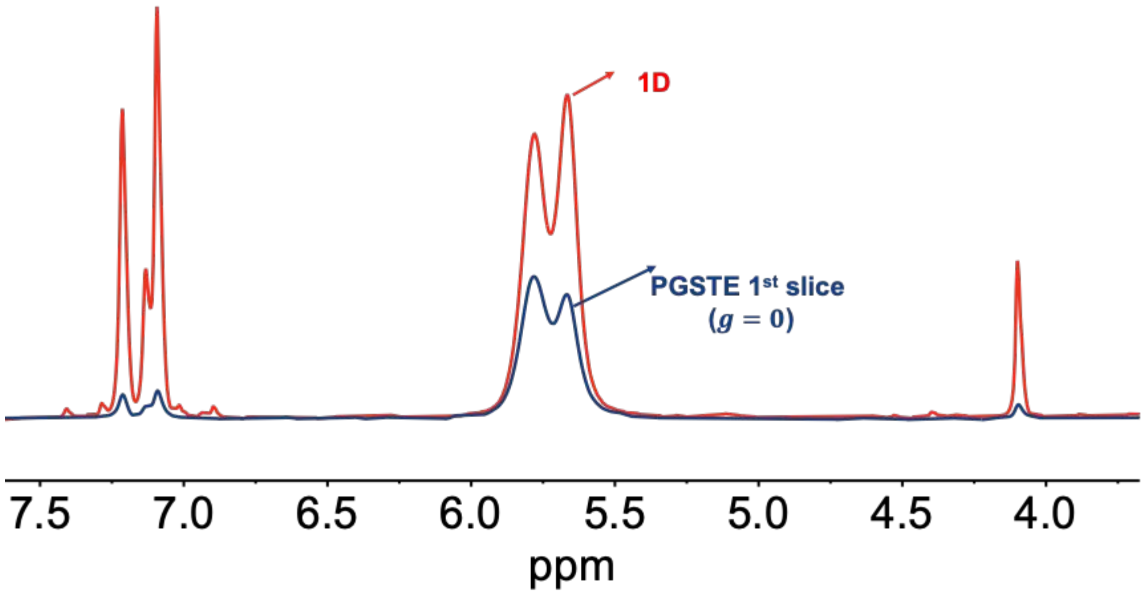

Figure S18: ^{1}H NMR comparison of 1D pulse (red) and PGSTE first slice (blue) for an OA-capped PbS QD solution titrated with 7.3 mM of free OAH. The difference in signal intensity arises from variations in the number of scans (1D = 32 scans, PGSTE = 16 scans) and relaxation delay time (1D = 27 s, PGSTE = 2 s), rather than signal weighing down due to spin-spin (T_2) or spin-lattice (T_1) relaxation. Additionally, the unchanged $FWHM$ and chemical shift indicate slow exchange ($\gg 25\ ms$) between $S - Bound$ OA and $free$ OAH.

14. ^{1}H NMR Spectrum Comparison of Acidic Proton with Varying Titration Concentrations and Temperature

We analyzed the chemical shift of the acidic proton in the presence of PbS QDs as a function of varying OAH titration concentration and temperatures, as shown in **Figures S19** and **S20**, respectively. For pure OAH in toluene-d_8, the acidic proton signal appears near 13 ppm with a full width at half maximum (FWHM) of 150 Hz (**Figure S19**). For OAH that is instantaneously weakly bound with QDs, slower tumbling of the particle along with ^{1}H dipole-dipole and/or chemical shift

anisotropy spin interactions will cause significant broadening of the acidic proton signal, making it difficult to resolve the signal from 1D ^{1}H NMR spectra.

Upon titrating excess OAH into a solution containing 24 mM of oleate (OA) bound to PbS QDs, we observed both a change in chemical shift and a broadening of the acidic proton peak, consistent with ligand exchange between the free and weakly bound OAH states. For example, in a sample containing PbS QDs titrated with 27.2 mM of excess OAH, the concentrations of free and weakly bound OAH is 25.8 mM and 9.4 mM, respectively. In this case, over 70% of the acidic proton signal arises from free OAH, causing the observed signal to appear closer to the chemical shift of the free ligand as shown in **Figure S19**. Conversely, in a sample titrated with only 7.3 mM OAH, the concentrations of free and weakly bound OAH is 3.2 mM and 8.2 mM, respectively. Here, the majority of the acidic proton signal (>70%) originates from the weakly bound OAH state, resulting in a significantly broader signal.

We further analyzed the sample containing PbS QDs titrated with 14.3 mM of excess OAH at temperatures ranging from 23 to -10 °C as shown in **Figure S20**. As the temperature decreases, the fraction of weakly bound OAH increases relative to free OAH (see **Figure 5B** in the manuscript). One might expect this to result in a broader acidic proton signal due to increased contributions from weakly bound species. However, we observed a pronounced increase in the intensity of acidic proton signal, with the peak gradually approaching 13 ppm. This behavior arises because the exchange rate between the free and weakly bound states become slower than the NMR timescale of measurement (inversely proportional to chemical shift difference between two states) at lower temperatures, resulting in the appearance of two distinct acidic proton signals. This trend was consistent across all titration concentrations examined. These changes in the acidic proton signal as a function of titration concentrations and temperatures further support our hypothesis that

the acidic head group (-COOH) participates in ligand binding to the QD surface *via* L-type coordination.

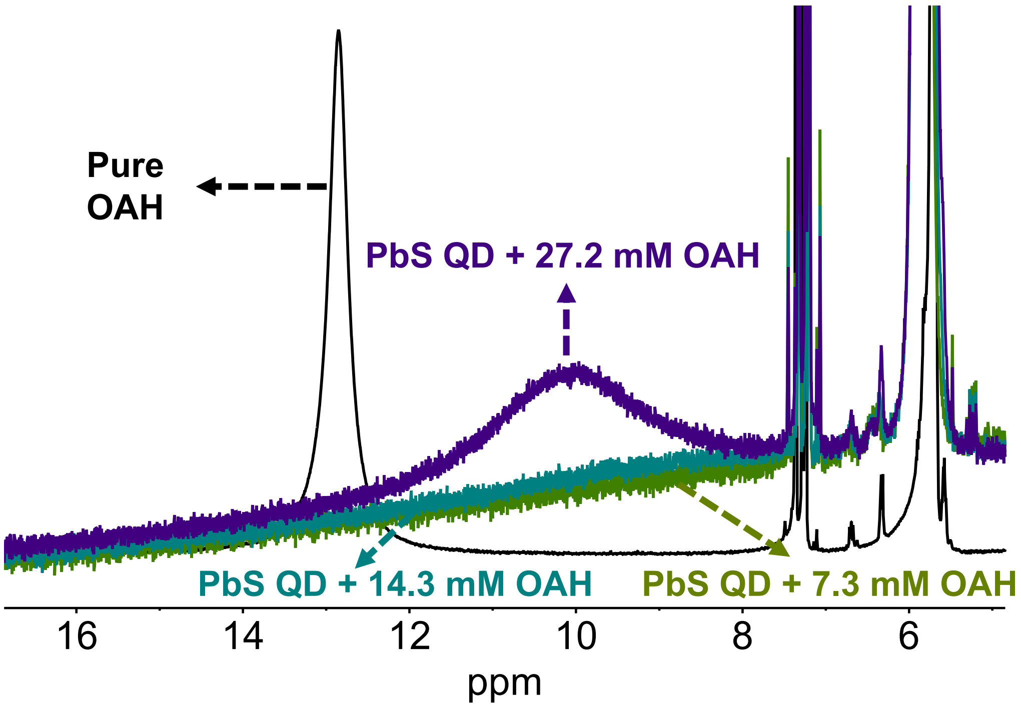

Figure S19: Chemical shift analysis of the acidic proton as a function of excess oleic acid (OAH) titration concentration. The control sample of pure (free) OAH in the toluene-d_8 provides a reference for chemical shift (13 ppm) and linewidth (150 Hz) of the acidic proton. Broadening and upfield shifting of the acidic proton signal increases with decreasing amount of OAH titrated into solution due to a larger population fraction of ligands (and acidic protons) associating with the QD surface.

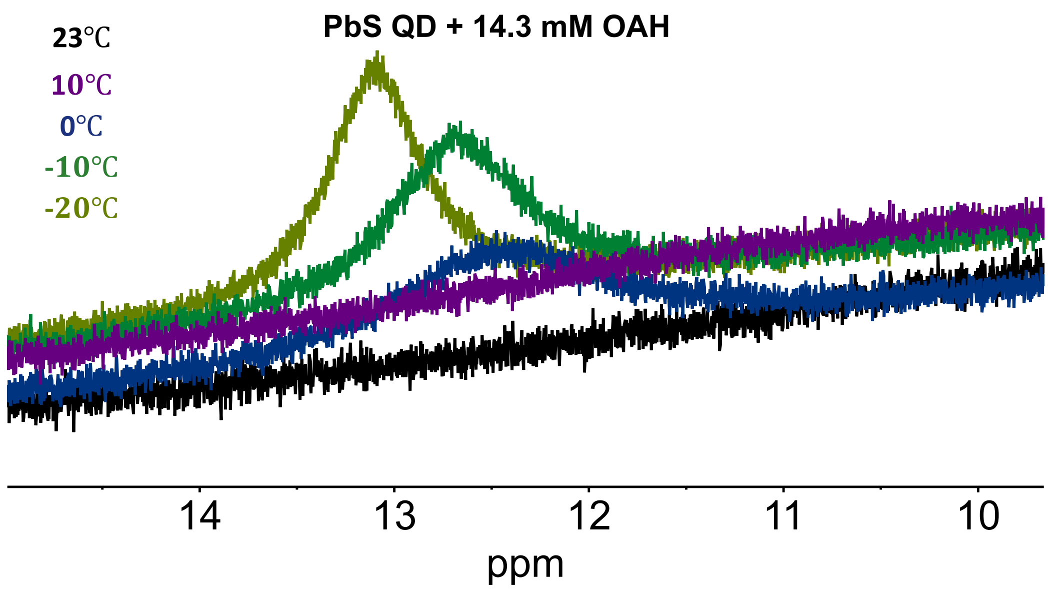

Figure S20: Temperature-dependent linewidth analysis of the acidic proton for a sample containing PbS QDs titrated with 14.3 mM of excess oleic acid OAH. At higher temperatures, the exchange rate falls within the intermediate exchange regime, resulting in significant broadening of the acidic proton signal (even to the extent of appearing as a flat baseline). At lower temperatures, the exchange rate falls within the slow exchange regime, leading to the appearance of two distinct acidic proton signal: one peak is narrow and near the chemical shift of free OAH (~ 13 ppm), and another broad proton signals whose chemical shift is unresolvable due to extensive line broadening.

References

(1) Moreels, I.; Lambert, K.; Smeets, D.; De Muynck, D.; Nollet, T.; Martins, J. C.; Vanhaecke, F.; Vantomme, A.; Delerue, C.; Allan, G.; Hens, Z. Size-Dependent Optical Properties of Colloidal PbS Quantum Dots. *ACS Nano* **2009**, *3*, 3023–3030.

(2) Costigliola, L.; Heyes, D. M.; Schrøder, T. B.; Dyre, J. C. Revisiting the Stokes-Einstein Relation without a Hydrodynamic Diameter. *J. Chem. Phys.* **2019**, *150*, 021101.

(3) Santos, F. J. V.; Nieto De Castro, C. A.; Dymond, J. H.; Dalaouti, N. K.; Assael, M. J.; Nagashima, A. Standard Reference Data for the Viscosity of Toluene. *J. Phys. Chem. Ref. Data* **2006**, *35*, 1–8.

(4) Kessler, M. L.; Dempsey, J. L. Mapping the Topology of PbS Nanocrystals through Displacement Isotherms of Surface-Bound Metal Oleate Complexes. *Chem. Mater.* **2020**, *32*, 2561–2571.

(5) Valdez, C. N.; Schimpf, A. M.; Gamelin, D. R.; Mayer, J. M. Low Capping Group Surface Density on Zinc Oxide Nanocrystals. *ACS Nano* **2014**, *8*, 9463–9470.

(6) Zherebetskyy, D.; Scheele, M.; Zhang, Y.; Bronstein, N.; Thompson, C.; Britt, D.; Salmeron, M.; Alivisatos, P.; Wang, L.-W. Hydroxylation of the Surface of PbS Nanocrystals Passivated with Oleic Acid. *Science* **2014**, *344*, 1380–1384.
(7) Aguilar, J. A.; Nilsson, M.; Bodenhausen, G.; Morris, G. A. Spin Echo NMR Spectra without J Modulation. *Chem. Commun.* **2012**, *48*, 811–813.
